# Digitoxin Inhibits Epithelial-to-Mesenchymal-Transition in Hereditary Castration Resistant Prostate Cancer

**DOI:** 10.3389/fonc.2019.00630

**Published:** 2019-08-02

**Authors:** Bette S. Pollard, Mark. A. Suckow, William R. Wolter, Joshua M. Starr, Ofer Eidelman, Clifton L. Dalgard, Parameet Kumar, Sharmistha Battacharyya, Meera Srivastava, Roopa Biswas, Matthew D. Wilkerson, Xijun Zhang, Qingfeng Yang, Harvey B. Pollard

**Affiliations:** ^1^Silver Pharmaceuticals, Rockville, MD, United States; ^2^Lobund Institute, University of Notre Dame, Notre Dame, IN, United States; ^3^Department of Anatomy, Physiology and Genetics, Uniformed Services University School of Medicine-America's Medical School, Uniformed Services University of the Health Sciences, Bethesda, MD, United States; ^4^Collaborative Health Initiative Research Program, Bethesda, MD, United States

**Keywords:** prostate cancer, hereditary, digitoxin, TGFβ, NFκB, TGFBR2, HSPB1

## Abstract

Castration Resistant Prostate Cancer (CRPC) is thought to be driven by a collaborative mechanism between TNFα/NFκB and TGFβ signaling, leading to inflammation, Epithelial-to-Mesenchymal-Transition (EMT), and metastasis. Initially, TGFβ is a tumor suppressor, but in advanced metastatic disease it switches to being a tumor promoter. TGFBR2 may play a critical role in this collaboration, as its expression is driven by NFκB and it is the primary receptor for TGFβ. We have previously reported that the cardenolide drug digitoxin blocks TNFα/NFκB-driven proinflammatory signaling. We therefore hypothesized that digitoxin might break the collaborative process between NFκB and TGFβ by also inhibiting expression of TGFBR2. We therefore tested whether TGFβ-driven EMT and resulting metastases would be suppressed. Here we show, *in vitro*, that digitoxin inhibits NFκB-driven TGFBR2 expression, as well as Vimentin, while elevating E-cadherin expression. Digitoxin also significantly reduces HSPB1 mRNA and the HSPB1/RBFOX2 mRNA ratio in PC3 cells. *In vivo*, in a syngeneic, immune competent rat model of metastatic CRPC, we show that digitoxin also suppresses *Tgfbr2* expression, as well as expression of other genes classically driven by NFκB, and of multiple EMT genes associated with metastasis. Concurrently, digitoxin suppresses tumor growth and metastasis in these animals, and prolongs survival. Gross tumor recurrence following tumor resection also appears prevented in *ca* 30% of cases. While the existence of a collaboration between NFκB and TGFβ to drive EMT and metastasis has previously been appreciated, we show here, for the first time, that chronic, low concentrations of digitoxin are able to block CRPC tumor progression, EMT and the ensuing metastatic disease.

## Introduction

Metastatic Castration Resistant Prostate Cancer (CRPC) is driven by a collaborative mechanism between NFκB-*driven* inflammation (1–3) and TGFβ-*driven* Epithelial-to-Mesenchymal-Transition (EMT) (4–6). EMT is a principal mechanism for metastasis, and thus has been a candidate target for cancer prevention and therapy. Currently, however, there are no approved anti-EMT drugs for any cancer (7). TGFβ has often been described as switching from initially being a tumor suppressor to being a tumor promoter in advanced metastatic disease (8, 9). In the case of metastatic CRPC, TGFβ has become such a tumor promoter (10, 11). EMT dependence on the collaboration between NFκB and TGFβ signaling is manifest both in wound healing (12), and in cancer (13, 14). The collaborative mechanism may also involve TGFBR2, the principal receptor for TGFβ. Not only does the TGFBR2 promoter include two functional NFκB binding sites, but severe metastatic disease can be suppressed by inhibiting TGFβ (15). For example, in mouse models of metastatic prostate cancer, soluble TGFBR2 has been deployed to trap circulating TGFβ and thus suppress tumor progression (4, 16).

Historically, the TNFα/NFκB pathway has been considered to be the “major culprit” contributing to the proinflammatory condition in prostate and other cancers (1, 3, 17, 18). However, the specific NFκB inhibitors for prostate and other cancers that have been tested clinically have met with profoundly unacceptable side effects, including nephrotoxicity and neurotoxicity (19). Furthermore, multiple rare loss-of-function mutations in genes affecting or affected by NFκB have been found to involve a broad range of abnormalities including impaired innate and acquired immune responses and defects of the central nervous system (19, 20). Given these problems, there has therefore been increasing pre-clinical interest in developing TGFβ and TGFBR2 inhibitors for prostate and other cancers (4, 5, 21). However, cancer cells have been shown to eventually be able to escape from suppression by a TGFBR2 knockout, thus indicating that additional strategies are needed to simultaneously inhibit TGFBR2's “backup partners” such as ERK1/2 (10). Inasmuch as TNFα/NFκB and TGFβ seem to be mutually interactive “culprits” in driving EMT, we have reasoned that it could be advantageous if they could be addressed simultaneously through the collaborative mechanism.

Our approach to developing an EMT therapeutic for CRPC has been to focus on the chemical biology of digitoxin, a cardenolide drug which we previously reported to potently block TNFα/NFκB signaling (22–25). Based on this property, we hypothesized that digitoxin might break the collaborating process between NFκB and TGFβ by inhibiting expression of TGFBR2. Our rationale was that by reducing the EMT process, we might be able to suppress CRPC growth and metastasis. *In vitro* studies shown below support this hypothesis. As an *in vivo* platform to test this hypothesis we deployed the Prostate Adenocarcinoma III (PA-III) rat model, a hereditary, spontaneous/autochthonous, transplantable, immunologically intact, castration resistant prostate cancer (CRPC) model in the Lobund-Wistar (LW) rat (26–29). The *in vivo* data, to be described below, clearly demonstrate that digitoxin can not only profoundly reduce the frequency and size of both primary tumors and metastases, but also reduce NFκB-driven inflammation, inhibit TGFBR2, reduce EMT, and increase survival. This is the first report to show that chronically administered digitoxin breaks the collaboration between NFκB and TGFβ, thus blocking tumor progression, EMT and ensuing metastatic CRPC disease.

## Materials and Methods

### Animals and PAIII Tumor Cells

The PAIII tumor has been maintained since 1976 by serially passaging freshly isolated tumor cells in naïve Lobund-Wistar (LW) rats. The tumor was originally isolated from a spontaneous (autochthonous), hormone resistant prostate tumor growing in an immunologically intact CRPC-prone Lobund-Wistar (LW) rat (26–30). Prior to implantation, dissociated PAIII tumor cells were maintained in a medium of DMEM high glucose medium (Invitrogen, Carlsbad, CA), supplemented with 10% Fetal Bovine Serum (FBS), and 1% penicillin-streptomycin solution, in 5% CO_2_, at 37°C.

### Protocols for Injection of PAIII Cells Into LW Rats

#### Protocol #1: Primary Tumor and Metastases Procedure

An equal number of viable PAIII cells (1 × 10^6^ cells) from an existing PAIII tumor, as determined by a trypan blue exclusion test, were injected subcutaneously (s.c.) into the right flank of thirty 2-month old LW rats (*ca*. 250 g body weight). After ten (10) days, 15 of the rats began to receive daily doses of digitoxin (0.03 mg/kg). Digitoxin (Sigma) was prepared as follows: (i) a stock solution was prepared by dissolving 300 mg digitoxin in 10 ml of 95% ethanol. (ii) two serial 1:20 dilutions were prepared by mixing, in order, 1 ml of the stock solution with 19 ml PBS. (iii) prorated volumes of the last dilution, calculated according to the specific weight of each rat, were then administered intraperitoneally (i.p.) each day in the 9–10 AM time interval. On the morning of day 40, all animals were sacrificed. The weight of each primary tumor was recorded. The number and diameter of pleural metastases were determined using a digital calipers (Control Company, Friendswood, TX). Primary tumors, and selected lung metastases, were immersed in liquid nitrogen, and stored for later analysis at minus 80°C. Over the course of this protocol, rats were observed daily by a veterinarian, board certified in laboratory and animal medicine, and no differences in body weight, appetite or physical appearance were noted either during or at the end of the experiment.

#### Protocol #2: Tail Vein Injection of Tumor Cells

An equal number of viable PAIII cells (1 × 10^6^ cells), as determined by a trypan blue exclusion test, were injected intravenously through the tail vein of thirty 2-month old LW rats (250 g body weight). Fifteen of the rats immediately began to receive daily injections (i.p) of digitoxin (0.03 mg/kg), as described and justified for Protocol #1. Lungs were harvested for analysis on day 10.

#### Protocol #3: Kaplan-Meier Analysis for Tumor Recurrence Following Tumor Resection

The strategy followed was based on a conventional clinical prostate cancer model for survival following therapy-dependent survival surgery (31). In this case, the tumor cells were implanted subcutaneously into 30 rats according to Protocol #1. Ten days later digitoxin treatment was initiated in 15 rats. Eight days later, tumors were removed surgically. Rats originally being treated with digitoxin continued to be treated (0.03 mg/kg/day) for 80 days.

### Measurement of Metastases

Lungs were removed and inflated with Bouin's Solution and placed in a jar containing the same solution. After overnight fixation, the Bouin's Solution was changed to 70% ethanol. The number and diameter of all detectable (diameter ≥0.1 mm) metastatic sites on the pleural surfaces were measured with digital calipers (Control Company, 308 West Edgewood Friendswood, TX 77546). Diameters of pleural metastases were measured in millimeter mode. The largest linear dimension was recorded as the diameter of the metastatic site.

### ELISA Analysis of Cytokines and Chemokines

Rat serum samples were analyzed on duosets from R&D Systems for rat serum IL-6 and CINC-1 (rat equivalent of human IL-8). Human cytokines and chemokines in serum or culture fluids were measured by multiplexed electro-chemiluminescence on the MSD Sector 6000 platform (MesoScale, Gaithersburg, MD), as described (32).

### Cells and Drugs

PC3 cells were obtained from ATCC, and were cultured in Hams F-12K nutrient mixture medium with 10% FBS and Pen-strep. HeLa cells were obtained from the ATCC, and cultured in Dulbecco's modified Eagle's medium, supplemented with 10% fetal bovine serum, 2 mM glutamine, penicillin (100 U/ml), and streptomycin (100 mg/ml). Digitoxin was obtained from Sigma (>95% purity). Oleandrin was obtained from Indofine Chemical (Hillsborough, NJ; >98% purity). Drugs were solubilized as stock solutions in 95% ethanol and diluted for experiments to a final ethanol concentration of 0.01%.

### Reporter Gene Assays

PC3 cells and HeLa cells were seeded in 6 well plates overnight, then cotransfected overnight (16 h) with NFkB-luc and LacZ plasmids using the Fugene HD transfection reagent (Roche). The cells were then treated with either digitoxin or oleandrin at different concentrations, for 12 h. Then cells were treated with TNFα, and the digitoxin and other cardiac glycosides, at different concentrations, for another 10 h. Cells were harvested and lysed with 1x passive lysis buffer. Luciferase assays were performed with the Promega Luciferase Assay System. The luciferase values were normalized to β-galactosidase activity.

### Western Blot Analysis for EMT Genes

PC3 cells and HeLa cells were treated with digitoxin at different concentrations and/or 20 ng/ml TNFα for 24 h. The medium was then replaced, including with fresh drugs and TNFα, and continuously treated for 3 days. Cells were then lysed in M2 buffer (20 mM pH 7.0 Tris, 0.5% NP-40, 250 mM NaCl, 3 mM EDTA, 3 mM EGTA, 2 mM dithiothreitol, 0.5 mM phenylmethyl- sulfonyl fluoride, 20 mM β-glycerol phosphate, 10 mM 4-Nitrophenyl phosphate disodium salt, 1 mM sodium vanadate, and 1 mg/ml of leupeptin). Twenty microgram of protein from the cell lysate of each sample was fractionated by SDS-PAGE and immunoblotted. The blots were visualized with chemiluminescent substrate (Pierce). The antibodies against Vimentin (code: sc-7870) and E-cadherin (code: sc-5565) were obtained from Santa Cruz Biotechnology, CA. Alternatively, cells were analyzed by capillary western blot (Protein Simple, San Jose, CA), as described (33).

### MTT Assays

Cells were seeded in 24 well plates at densities of *ca*. 10^4^/ml, and then treated with digitoxin at the different concentrations for 24 h. Fresh medium and drug were added every day. Cells were continuously treated in this manner for up to 3 days (HeLa cells) or up to 6 days (PC3 cells). Then 1/10 volume 12 mM MTT stock solution was added. After 4 h incubation at 37°C, an equal volume of 10% SDS solution was added to the wells. Absorbance was read at 570 nm after another 4 h incubation.

### Transcription Profiling and Sequence Analysis

Samples of grossly dissected, frozen (−80°C) primary tumors were mounted on a cryostat chuck. Thin sections (20 micron) were cut and stained with Hematoxylin and Eosin (H& E) to distinguish between vital from non-vital areas of the tumors. Serial thick sections (300 micron) were then cut and mounted on sub-0°C slides. Using the 20 micron stained slides as a “stencil,” ten random samples were micro-dissected from viable regions of the tumor in 300 micron thick sections with a 1 mm diameter micropunch device. The tumor samples were then soaked in RNA Later at 4°C overnight.

#### Isolation and Analysis of Messenger RNAs

Total RNA isolated from micro dissected tissues was validated for integrity using the Experion Automated Electrophoresis System (BioRad) before library preparation. An amount of 1 μg total RNA was input for library preparation using the TruSeq RNA Sample Prep Kit v2 (Illumina). Sequencing libraries were validated for size and integrity using an Experion Automated Electrophoresis System (BioRad), and library concentration was determined using the KAPA Library Quantification Kits for Illumina (KAPA Biosystems). Twelve to fifteen pmole of Library were used as input for clustering by a c-Bot (Illumina) before sequencing on a Genome Analyzer IIx (Illumina). Sequencing was performed by single read of 101 bp length with an indexing read of 7 bp. FASTQ data were generated using CASAVA, and raw reads were analyzed and groomed using FASTQC and FASTQ Groomer, respectively. RPKM values for transcriptome expression levels were generated using Bowtie 2, TopHat 2, and Cufflinks 2. RPKM values were used for Comparative Marker Selection and differential gene analysis by Cuffdiff. Raw FASTQ files for each profiled sample and processed data of FPKM values for transcript expression levels are available at the NCBI Gene Expression Omnibus at the accession number GEO133696.

#### Isolation and Analysis of microRNAs

The tissues were homogenized with RNA lysis buffer (Ambion) according to the manufacturer's protocol. Multiplex Reverse Transcription was performed with the TaqMan MicroRNA Reverse Transcription Kit (Applied Biosystems). For microRNA analysis, the miRNA expression profiles were analyzed by the TaqMan Low Density Rodent MicroRNA Panel v2, as described (34). Briefly, multiplex Reverse Transcription was performed with TaqMan MicroRNA Reverse Transcription Kit (Applied Biosystems). Following reverse transcription, each RT reaction was diluted and mixed with TaqMan Gene expression Master Mix (2X). One hundred microliter of the RT reaction-specific PCR reaction mix was loaded into the corresponding fill ports of the TaqMan Low Density Rodent MicroRNA Panel. Processed miRNA expression data were analyzed using the Ingenuity Pathways Analysis (IPA) algorithm.

### Bioinformatics and Statistics

MicroRNA and mRNA relationship analysis was generated using overlap between the TargetScan (Release 5.1, Whitehead Institute) predicted miR targets and the RNA seq data (Illumina GIIx). The IPA algorithm was also used to generate networks focusing on prostate cancer, and metastases. Parametric (Student *t*-test) and non-parametric (Mann-Whitney) tests were used to compare tumor weights, number and sizes of classical metastases, following subcutaneous flank injection of cells. Identical tests were performed to analyze number and size of metastasis-like lung foci following intravenous cell administration. Histograms were prepared on the basis of log distributions of diameters of tumor-seeded metastases, and metastasis-like lung foci following intravenous administration. Unbiased averages, means and medians of distributions were prepared using total data. Analyses of cytokine and chemokine data were analyzed using GraphPad, Version 6. In all cases, differences were taken as significant if *P* < 0.05.

## Results

### NFκB Signaling and EMT Gene Expression in Human Prostate Cancer PC3 Cells

**Figure 1:**
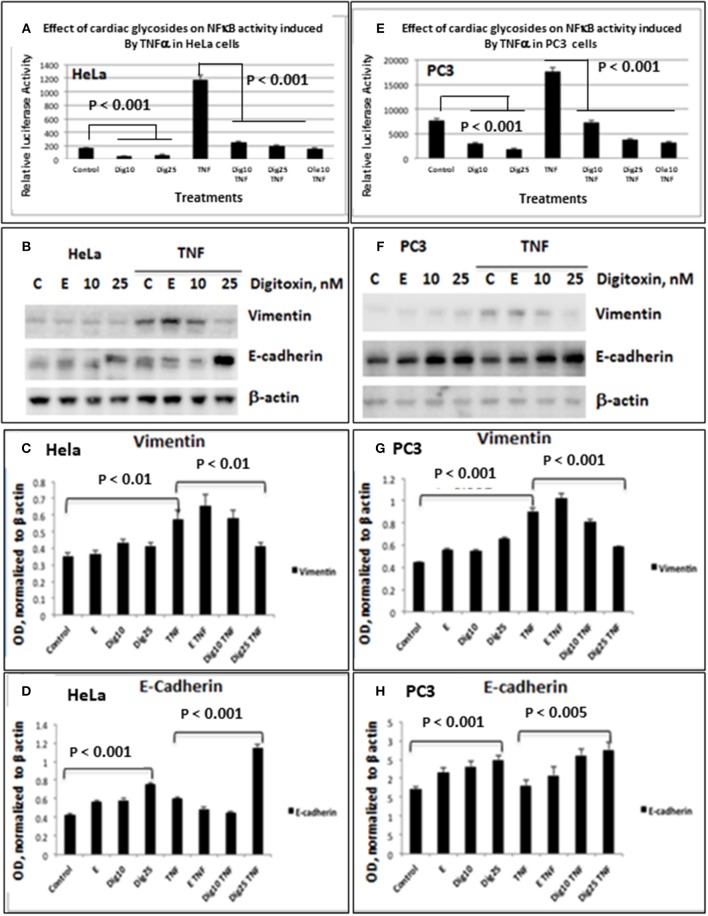
Effects of low dose digitoxin on NFκB activation and Epithelial-Mesenchymal Transition (EMT) protein expression in HeLa and PC3 cells. **(A)** Effect of digitoxin on baseline and TNFα-activated NFκB activation in HeLa cells. HeLa cells were co-transfected for 16 h with NFκB-luc and LacZ plasmids using the Fugene HD transfection reagent. Thereafter the cells were treated for 12 h with different concentration of digitoxin, and then treated with digitoxin and 20 ng/ml TNFα for 12 subsequent hours. Luciferase assays were performed with the Promega Luciferase Assay System, and the luciferase values normalized to β-galactosidase (LacZ) activity. This experiment is representative of three independent experiments. **(B)** Western blot of digitoxin effects on baseline and TNFα activated expression of Vimentin and E-cadherin in HeLa cells. Hela cells were treated with digitoxin at 10 and 25 nM concentrations, and either 20 ng/ml TNFα or control, and incubated for 24 h. The media, with drug and TNFα, were replaced every 24 h, for a total of 3 days. After this 3 day experiment, cells were harvested and assayed by western blot analysis for Vimentin and E-cadherin. Digitoxin reduced Vimentin expression, but raised E-cadherin expression. This experiment is representative of three independent experiments. **(C)** Bar graph of digitoxin effects on baseline and TNFα activated expression of Vimentin in HeLa cells. Western blots were scanned, and specific proteins were normalized with respect to β-actin expression levels. Vimentin levels were unaffected under control conditions. Exposure to TNFα raised Vimentin levels significantly (*p* < 0.01). Twenty five nanomolar digitoxin reduced TNFα-induced Vimentin expression significantly (*P* < 0.01). **(D)** Bar graph of digitoxin effects on baseline- and TNFα-activated expression of E-cadherin in HeLa cells. Western blots were scanned, and specific proteins were normalized with respect to β-actin expression levels. Under control conditions, 25 nM digitoxin raised levels of E-cadherin (*P* < 0.001). Following treatment of cells with TNFα, 25 nM digitoxin significantly, raised levels of E-cadherin even further (*P* < 0.001). **(E)** Effect of digitoxin on baseline and TNFα-activated NFκB activation in PC3 cells. PC3 cells were co-transfected for 16 h with NFκB-luc and LacZ plasmids using the Fugene HD transfection reagent. Thereafter the cells were treated for 12 h with different concentration of digitoxin, and then treated with the digitoxin and 20 ng/ml TNFα for 10 subsequent hours. Luciferase assays were performed with the Promega Luciferase Assay System, and the luciferase values normalized to β-galactosidase (LacZ) activity. This experiment is representative of three independent experiments. **(F)** Western blot of digitoxin effects on baseline and TNFα activated expression of Vimentin and E-cadherin in PC3 cells. PC3 cells were treated with digitoxin at 10 and 25 nM concentrations, and either 20 ng/ml TNFα or control, and incubated for 24 h. The media, with drug and TNFα, were replaced every 24 h, for a total of 3 days. After this 3 day experiment, cells were harvested and assayed by western blot analysis for Vimentin and E-cadherin. This experiment is representative of three independent experiments. **(G)** Bar graph of digitoxin effects on baseline and TNFα activated expression of Vimentin in PC3 cells. Western blots were scanned, and specific proteins were normalized with respect to β-actin expression levels. Vimentin levels were unaffected under control conditions. Exposure to TNFα raised Vimentin levels significantly (*p* < 0.01). Twenty five nanomolar digitoxin reduced TNFα-induced Vimentin expression significantly (*P* < 0.001). **(H)** Bar graph of digitoxin effects on baseline and TNFα activated expression of E-cadherin in PC3 cells. Western blots were scanned, and specific proteins were normalized with respect to β-actin expression levels. Under control conditions, 25 nM digitoxin raised levels of E-cadherin (*P* < 0.001). Following treatment of cells with TNFα, 10 nM and 25 nM digitoxin progressively raised E-Cadherin levels, with highest significance for the 25 nM digitoxin treatment *P* < 0.005).

As shown for HeLa cells (Figure 1A) and PC3 cells (Figure 1E), baseline and TNFα-activated NFκB promoter activities are profoundly reduced by low concentrations of digitoxin. We included parallel HeLa cell experiments to evaluate the possible generality of digitoxin action. Additionally, low concentrations of the digitoxin cardenolide analog oleandrin effectively suppress TNFα/NFκB signaling in both cell types. We had previously reported that oleandrin, like digitoxin, was a potent suppressor of NFκB signaling (22). Thus, both cardenolide drugs contribute to reducing NFκB signaling in these cells, affecting both baseline and TNFα-dependent mechanisms.

**Figure 2:**
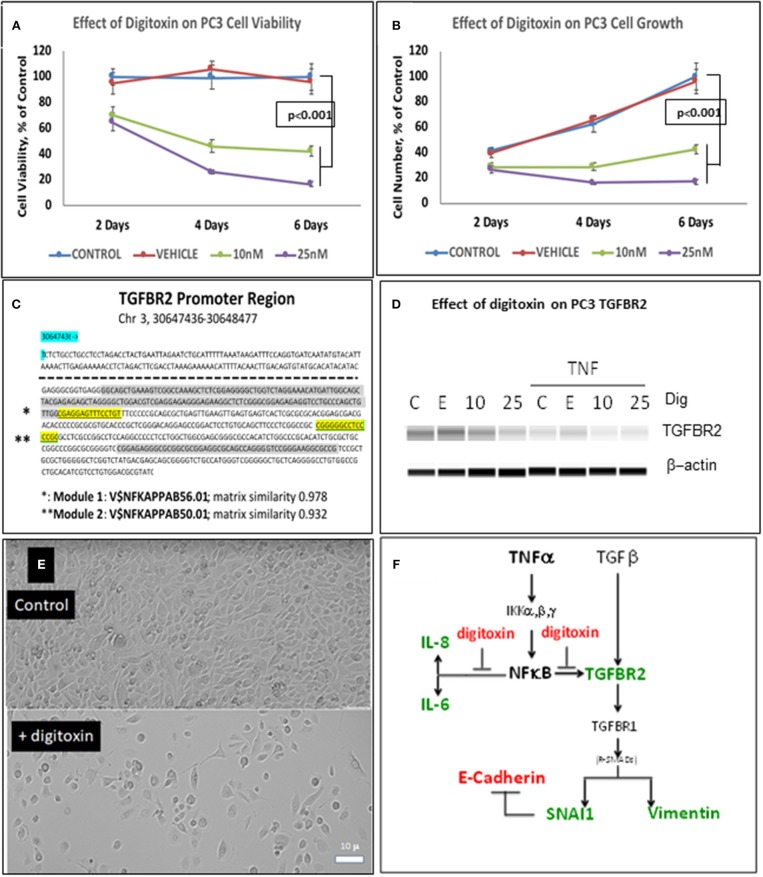
Influence of digitoxin on PC3 and Hela cell viability and growth, and on expression of TGFBR2 protein in PC3 cells. **(A)** Effect of digitoxin on PC3 cell viability. Conditions and abbreviations are as in part A. **(B)** Effect of digitoxin on PC3 cell growth. Conditions and abbreviations are as in part A. **(C)** TGFBR2 Promoter sequence analysis. Data are from the Genomatix Pathway System. Module 1 (^*^) is a composite tandem module of transcription factors composed of two KLFS (Krupple-like Transcription Factor sequences; ^*^ gray color code), followed by an NFκB family member p65 (RELA, ^*^ highlight). This is the “strongest” NFκB promoter. Module 2 (^**^) is the “less strong” NFκB site, and is juxtaposed to a Sp1 site (^**^ gray color code). Matrix Similarity is scored as 1.000 being 100% strength and 0.900 being 10% strength. For clarity, the dotted line represents sequences distal to proximal regions containing the two κB sequences. **(D)** Influence of digitoxin on TGFBR2 expression in PC-3 Cells. PC-3 cells were cultured for 3 days with TNFα (10 ng/ml), and either 0, 10 or 25 nM digitoxin. Fresh media, with TNFα and digitoxin, were added each day. Capillary Western blot analysis (Protein Simple, San Jose, CA), shown here, was carried out as described in Methods. **(E)** Influence of digitoxin on PC cell culture morphology. Upper panel are PC-3 cells growing under vehicle control conditions for 3 days. Lower panel are PC cells growing in the presence of 25 nM digitoxin. The size marker in lower left of the lower panel is 10 microns. As expected from analysis of growth there are far fewer cells surviving in the presence of digitoxin. In all cases, all analytic data are normalized to the MTT signal to correct for cell number. **(F)** Summary: Digitoxin blocks EMT collaboration between NFκB and TGFβ signaling by inhibiting expression of TGFBR2 in PC3 cells. Downstream of TGFBR2 there is reduced Vimentin expression and elevation of E- Cadherin due to inhibition of SNAI1. Color code: red, elevated; green, reduced.

To test whether low dose digitoxin affected expression of epithelial- mesenchymal-transition (EMT) proteins, we tested PC-3 and HeLa cells for digitoxin-dependent decreases in the EMT effector protein Vimentin and concomitant increases in the EMT effector protein E-Cadherin. The western blots for HeLa cells (Figure 1B) and for PC-3 cells (Figures 1F,G) show that concentrations of 10 and 25 nM digitoxin systematically and significantly *suppress* expression of TNFα-induced Vimentin. Simultaneously, the same conditions significantly and profoundly *elevate* E-Cadherin expression (Figures 1F,H). Summary bar graphs in Figure 1C (for HeLa cells) and Figure 1D (for PC-3 cells) show that digitoxin reversals of EMT signaling (*viz*., reduction of Vimentin; elevation of E-Cadherin) are dose dependent and statistically significant at a digitoxin concentration of 25 nM. These data indicate that concurrent TNFα treatment is needed for optimal activation of EMT activity. Consistently, digitoxin also reduces the viability (Figure 2A) and growth rate (Figure 2B) of PC3 cells. Figure 2C summarizes the TGFBR2 promoter sequence, emphasizing the proximal tandem NFκB binding sequences. Consistently, Figure 2D shows that 25 mM digitoxin optimally suppresses TGFBR2 protein expression in PC3 cells, in the presence or absence of concurrent TNFα treatment. Finally, Figure 2E shows that PC3 cells grow as fibroblast-like cells under control condition, and that digitoxin reduces PC3 cell density and may change structural appearance. In summary, Figure 2F shows that digitoxin blocks not only TNFα/NFκB signaling in PC3 cells, but also blocks signaling to TGFβ through TGFBR2, and subsequent EMT signaling through E-cadherin and Vimentin.

**Figure 3:**
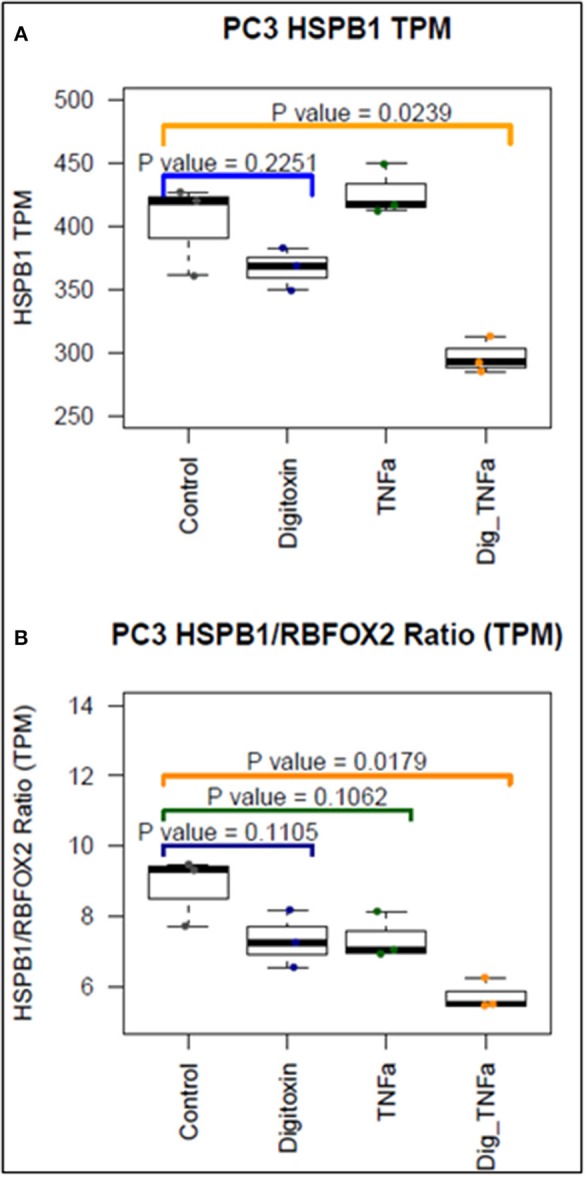
Effect of digitoxin on *HSPB1* and *RBFOX2* mRNA expression in PC3 cells. **(A)** Effect of digitoxin (25 nM) on *HSPB1* mRNA expression. Neither digitoxin (25 NM) nor TNFa (10 ng/ml) have significant effects on HSPB1 mRNA expression. However, both TNFa and digitoxin together significantly reduce HSPB1 expression (*P* = 0.0239; *N* = 3). Three day exposure; Data are from RNA-seq experiments. TPM are transcripts per million. **(B)** Effect of digitoxin (25 nM) on HSPB1/*RBFOX2* mRNA ratio. Exposure of cells to digitoxin alone, or TNFα alone, do not exert a significant effect in the ratio. However, in the presence of both TNFα and digitoxin, the ratio is significantly reduced by ca. 41% (*P* = 0.0179; *N* = 3). Three day exposure, Data are from RNA-seq experiments. TPM are transcripts per million.

To further test the ability of digitoxin to suppress EMT, we used RNA-seq to examine the effect of digitoxin on the expression and ratio of mRNAs for *HSPB1* and *RBFOX2*. HSPB1 is a positive biomarker for EMT in prostate cancer (35), while RBFOX2, regulates both epithelial and mesenchymal splicing events (36). Figure 3A shows that incubation of PC3 cells with digitoxin and TNFα for 3 days significantly results in a *ca* 30% reduction in *HSPB1* mRNA (*P* = 0.0239) compared to control conditions. Figure 3B shows that the ratio of *HSPB1/RBFOX2* is significantly reduced by *ca*. 41% (*P* = 0.0179). The digitoxin-dependent reduction of both HSPB1 and the HSPB1/RBFOX2 ratio can be interpreted as supporting the concept that digitoxin suppresses EMT in human PC3 cells.

### Digitoxin Suppresses Primary and Metastatic Prostate Cancer

**Figure 4:**
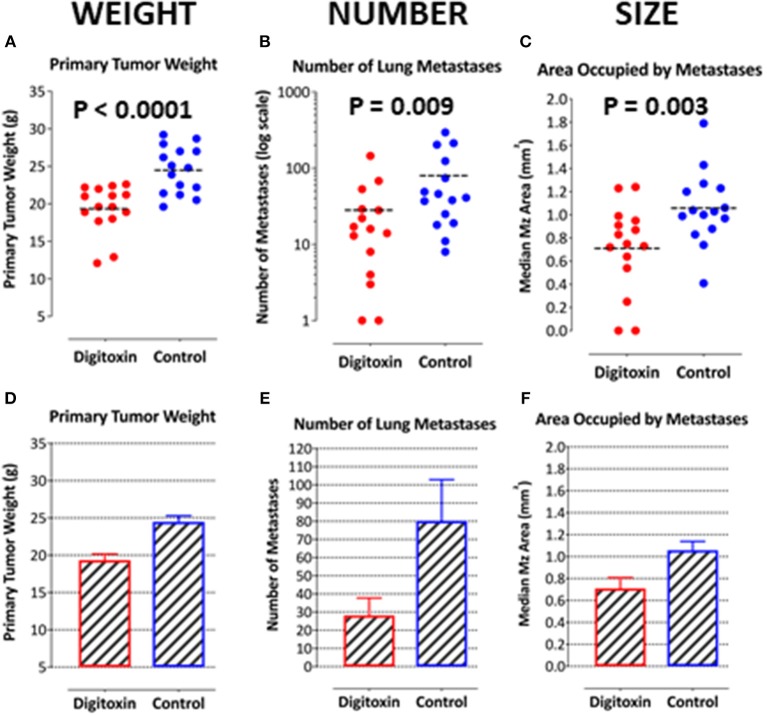
Influence of digitoxin treatment on primary tumor weight and on number and sizes of metastases from tumor cells implanted subcutaneously. **(A)** Digitoxin effect on primary tumor weight. The differences in digitoxin-dependent average and median weights vs. controls are significant (parametric Student's *t*-test, *p* = 6.4 × 10 ^−5^; non-parametric Mann-Whitney, *p* = 2 × 10^−4^). Data are summarized in Table 1, and as a bargraph in **(D)**. **(B)** Digitoxin effect on number of metastases, seeded from the primary tumor. Numbers of metastases are found to be reduced by digitoxin-treatment from an average of 80/lung to an average of 28/lung. The differences are significant (for the averages, parametric Student's *t*-test, *p* = 0.022; non-parametric Mann-Whitney, *p* = 0.009). Data are summarized in Table 1, and as a bar graph in **(E)**. **(C)** Digitoxin effect on sizes of metastases, seeded from the primary tumor. Digitoxin treatment was found to reduce the median area of metastases from 1.06 to 0.71 mm^2^. The differences were significant (parametric Student's *t*-test, *p* = 0.0001; non-parametric Mann-Whitney, *p* = 0.004). Data are summarized in Table 1, and as a bar graph in **(F)**. **(D)** Bar graph of data in **(A)**. Digitoxin effect on primary tumor weight. **(E)** Bar graph of data in **(B)**. Digitoxin effect on number of metastases, seeded from the primary tumor. **(F)** Bar graph of data in **(C)**. Digitoxin effect on sizes of metastases, seeded from the primary tumor.

**Figure 5:**
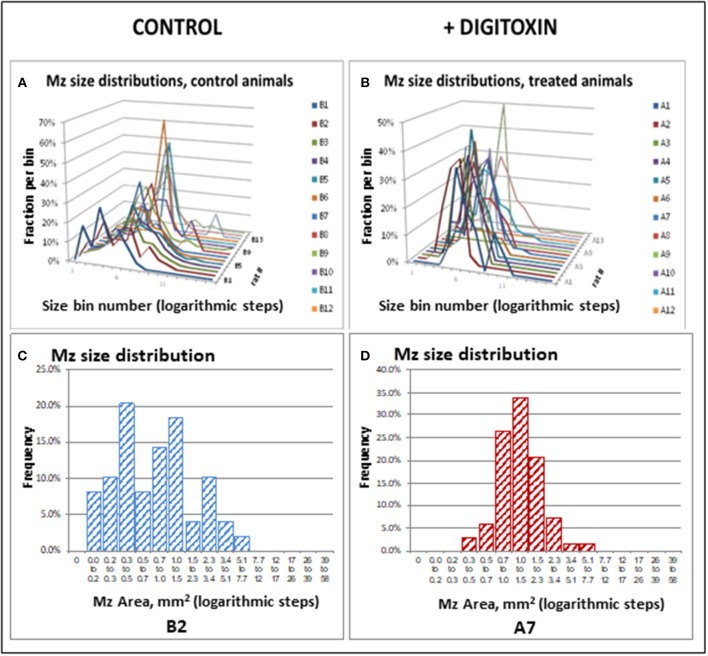
Stagger plots show effect of digitoxin on diameters of metastatic sites on pleural surface which have seeded from primary tumors. **(A)** Distribution of individual metastasis diameters in control (no drug treatment) rats. There are 15 such rats in the plot. **(B)** Distribution of individual metastasis diameters in treated (treatment with digitoxin) rats. There are 15 control rats in the plot. **(C)** Histogram of size distribution for metastases from a representative control rat Distribution is multimodal. **(D)** Histogram of size distribution for metastases from a representative digitoxin-treated animal. The distribution is monomodal.

To test for the ability of digitoxin to suppress castration resistant prostate cancer *in vivo*, we subcutaneously injected passaged rat tumor cells into syngeneic rats. Then, 10 days later we treating the rats with digitoxin or vehicle. After a total of 40 days we measured both growth of the primary tumor and metastases to the lung. As shown in Table 1 and Figures 4A–F, digitoxin treatment significantly reduced primary tumor weights. Digitoxin also reduced the numbers and sizes of metastases. Inasmuch as 10 days had passed between initial tumor cell implant and initiation of digitoxin treatment, the data suggested that metastasis was an ongoing process, and could have been interrupted by digitoxin. If this hypothesis were correct, we reasoned that the sizes of metastasis should be limited in the digitoxin-treated tumors. To test this hypothesis, we constructed a comprehensive stagger plot for drug-dependent changes in metastasis size. For this purpose 15 control (vehicle) treated rats (see Figure 5A) and 15 digitoxin-treated rats (see Figure 5B) were comprehensively analyzed. Inspection of controls in Figure 5A showed a wide distribution of sizes. However, in rats treated with digitoxin there was only a limited distribution of sizes (see Figure 5B). To quantitate this observation further, we prepared detailed histograms from one typical control rat (Figure 5C) and one typical digitoxin-treated rat (Figure 5D). The control rat had a multimodal distribution of sizes, while the digitoxin-treated rat revealed a virtual *unimodal* distribution of sizes. These data suggested that digitoxin might arrest seeding of metastases upon administration on day 10, and permit survival and limited growth only for those metastases that had already been established.

**Table 1:** Influence of digitoxin on growth of tumor and metastases, from cells administered by subcutaneous route in flank.

**Primary tumor given on day 0, digitoxin on day 10 for 30 days**	**Primary tumor**	**Mz number**	**Mz diameter, mm**		**Mz area, mm**^****2****^		**Total Mz Area**
			**Average**	**Median**	**Average**	**Median**	
**Digitoxin-treated (*****n*** **=** **15 rats)**							
Average	19.3	28	0.92	0.87	0.86	0.71	33
Median	19.6	16	1.07	0.98	0.96	0.75	16
SD	3.2	38	0.42	0.39	0.51	0.38	50
Sem	0.9	10	0.11	0.10	0.14	0.10	13
**Control (*****n*** **=** **15 rats)**							
Average	24.5	80	1.25	1.15	1.50	1.06	123
Median	24.8	41	1.24	1.15	1.41	1.03	55
SD	3.1	88	0.18	0.18	0.47	0.31	149
Sem	0.8	24	0.05	0.05	0.13	0.08	40
**Comparison**							
**Control vs. Digitoxin-treated**							
Ratio of averages	1.27	2.86	1.36	1.32	1.73	1.49	3.78
Ratio of medians	1.27	2.56	1.15	1.17	1.48	1.37	3.52
Student's *t*-test (1–tailed)	6.40E-05	0.022	0.004	0.010	0.001	0.005	0.017
Mann-Whitney test (1–tailed)	2.00E-04	0.009	0.002	0.004	0.001	0.004	0.002

**Figure 6:**
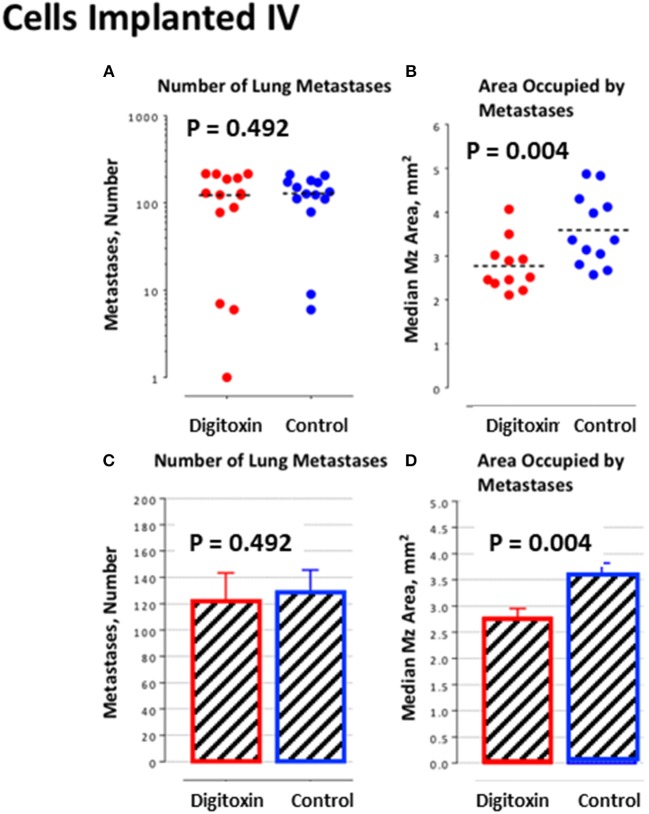
Influence of digitoxin treatment on number and size of lung metastases from tumor cells implanted intravenously. **(A)** Number of lung metastases following intravenous administration of tumor cells. Color code: red = digitoxin; blue vehicle control. Medians are identical *P*, 2 tailed = 0.492; *N*, digitoxin = 13; *N*, control = 14. **(B)** Area occupied by lung metastases following intravenous administration of tumor cells. Color code the same as in part A. Significantly less area occupied by tumor cells in digitoxin-treated rats (*P*, 2 tailed = 0.004; *N*, digitoxin = 11; *N*, control = 12). **(C)** Bar plot for data in Part A. **(D)** Bar plot for data in Part B.

To further test this hypothesis, we bypassed biological metastasis by administering tumor cells through intravenous tail vein injection. Digitoxin treatment was then initiated immediately, and continued until animals were sacrificed on day 10. Table 2 and Figures 6A,C) show that the *number* of implanted/metastatic sites were now virtually the same, with or without digitoxin. However, *sizes* of intravenous-administered metastases are now significantly lower in the drug-treated group (Figures 6B,D; *P* = 0.004). These data suggest that once in circulation, the cancer cells have equivalent access to the pleural niche. However, digitoxin also reduced the growth rate for the implanted metastasis, whether it has been seeded directly from the primary tumor, or by intravenous injection.

**Table 2:** Influence of digitoxin on implantation and growth of metastases-like tumors from cells administered by intravenous route.

**Prostate cancer cells and digitoxin both given on day 0 for 10 days**	**Primary tumor**	**Mz number**	**Mz diameter, mm**		**Mz Area, mm**^****2****^		**Total Mz Area**
			**Average**	**Median**	**Average**	**Median**	
**Digitoxin**-**treated (*****n*** **=** **14 rats)**							
Average	–	122	1.79	1.76	2.76	2.50	390
Median	–	127	1.80	1.77	2.72	2.46	327
SD	–	78	0.31	0.29	0.92	0.79	294
Sem	–	21	0.08	0.08	0.24	0.21	78
**Control (*****n*** **=** **14 rats)**							
Average	–	129	2.10	2.07	3.79	3.43	526
Median	–	131	2.07	2.07	3.53	3.37	498
SD	–	64	0.34	0.33	1.16	1.00	317
Sem	–	17	0.09	0.09	0.31	0.27	85
**Comparison**							
**Control vs. Digitoxin-treated**							
Ratio of averages	–	1.05	1.17	1.17	1.37	1.37	1.35
Ratio of medians	–	1.03	1.14	1.17	1.30	1.37	1.52
Student's *t*-test (1–tailed)	–	0.411	0.009	0.008	0.008	0.005	0.125
Mann-Whitney test (1–tailed)	–	0.492	0.005	0.003	0.005	0.003	0.104

**Figure 7:**
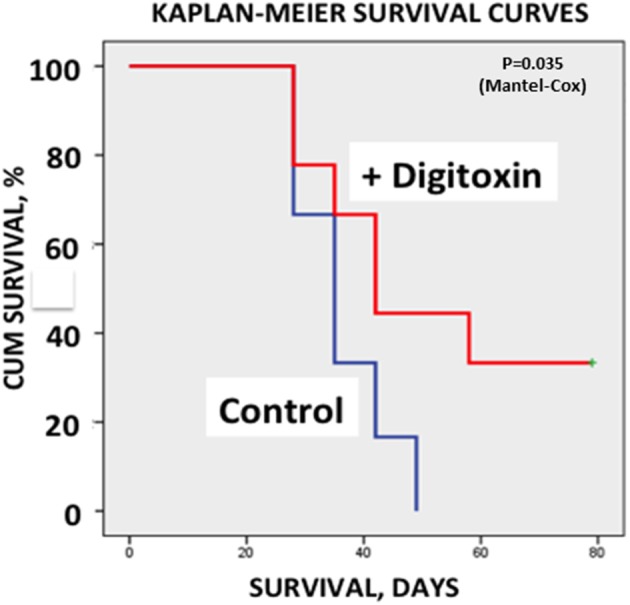
Influence of digitoxin treatment on survival following tumor resection. Following subcutaneous implantation of tumor cells into 30 rats, digitoxin administration was initiated on 15 of the rats 10 days later. Eight days late, all tumors were resected. Surviving digitoxin-treated rats received daily digitoxin treatment for duration of the experiment. All surviving control (no digitoxin treatment) rats experienced tumor recurrence and all had expired by *ca*. day 50. However, by day 80, 30% of digitoxin-treated rats were still surviving a grossly tumor free when necropsied. Kaplan-Meier Survival Curves were significantly different based on a log rank Mantel-Cox Chi-Square test (*p*, 2 tailed = 0.035).

Finally, to test whether treatment with digitoxin provided any survival benefits to the rat under a clinically relevant condition, we again implanted tumor cells subcutaneously into 30 rats. Ten days later, we began treating half the rats with digitoxin. Eight days later (day 18), we resected all primary tumors from all the animals. Digitoxin- treated rats which survived resection surgery continued to receive a daily injection of digitoxin for the duration of the experiment. Figure 7 shows that all control rats experienced primary tumor recurrence, and had expired by *ca* day 50. By contrast, digitoxin treated rats survived significantly longer, with 30% of the animals surviving up to day 80. Post-mortem analysis on day 80 of these drug-treated survivors showed no evidence of primary tumor recurrence after resection. Survival curves of treated vs. untreated were significantly different based on a log rank Mantel-Cox Chi-Square test (*P*, 2 tailed = 0.035). Thus, the effect of the drug treatment was to extend survival for digitoxin- treated rats following resection, and to confer long term resistance to tumor recurrence on a significant fraction of the digitoxin-treated animals following tumor resection.

### Tumor Stratification and Grouping for Transcription Profiling

**Figure 8:**
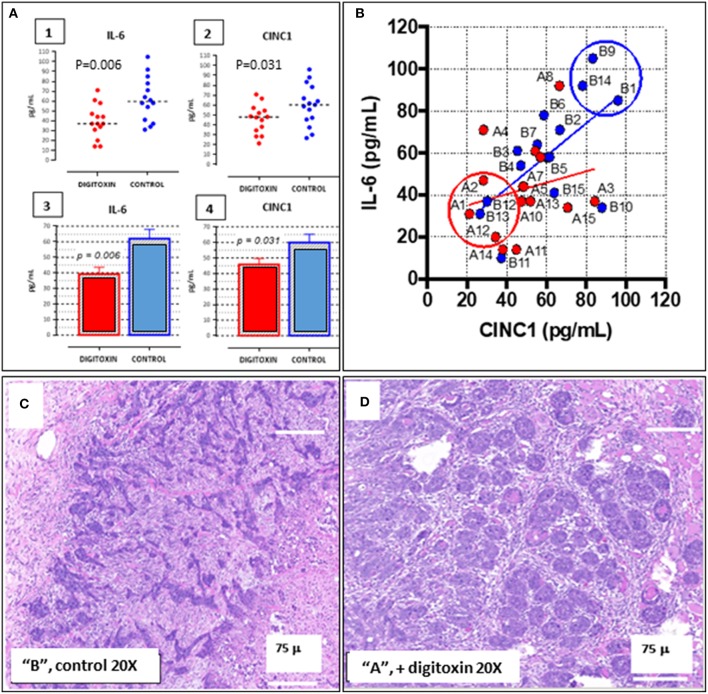
Distribution of IL-6 and CINC-1 (rat equivalent to human IL-8) in serum from drug-treated vs. untreated rats bearing CRPC tumors. **(A)** Dot-plots of serum cytokine and chemokine. (1) IL-6 in serum from rats bearing tumors, treated with digitoxin (red dots) or without digitoxin (blue dots). (2) Dot-plots of CINC-1 in serum from rats bearing tumors, treated with (red dots) or without (blue dots) digitoxin. (3) Bar graph of IL-6 data in part (1). (4) Bar graph of CINC-1 data in part (2). **(B)** Graph of levels of IL-6 and CINC-1 in sera from rats bearing tumors. Color code is treatment with digitoxin (red dots) or control (blue dots). **(C)** Representative Hematoxylin & Eosin-stained section of tumor from control (“B”) rat section. Cancer cells occur as “islands” of dark staining cells embedded in matrix. Magnification is 20×; Bar is 75 microns. **(D)** Representative Hematoxylin & Eosin-stained section of tumor from digitoxin-treated (“A”) rat. Tumor cells occur as “nests” of dark staining cells embedded in matrix. Magnification is 20×; Bar is 75 microns.

To aid in grouping and selecting tumors and tumor regions suitable for RNA-seq analysis, we first stratified animals on the basis of serum IL-6 and serum CINC-1 (see Figure 8A, 1–4). CINC-1 is the equivalent of human IL-8 in the rat. High levels of circulating IL-6 and IL-8 are biomarkers for severe metastatic prostate cancer in humans (37, 38), and relative levels of these immune mediators are considered to be surrogates for tumor burden (39, 40). Figure 8A, 1–4 shows that by day 30 after starting digitoxin administration, tumor-bearing rats had significantly *lower* levels of both IL-6 and CINC-1 in their sera. Thus, the composite *reductions* in IL-6 and CINC-1 correlate closely with the digitoxin-dependent *reductions* in primary tumor weights shown in Table 1. Figure 8B shows the two biomarkers graphed against one another. This strategy linearly stratifies control “B” rats, which express the highest levels of both biomarkers, from digitoxin-treated “A” rats, which express the lowest level of both biomarkers. “A” rats (digitoxin-treated) and “B” rats (vehicle only) were then **p**icked randomly from the lower and upper extremes, respectively, from the IL-6 vs. CINC-1 graph (Figure 8B). Frozen tumor tissues from these rats were then individually sectioned serially into 20 micron and 300 micron sections. The 20 micron sections were stained with Haemotoxylin and Eosin to permit easy identification of cancer cell islands. Representative stained sections are shown in Figure 8C for a control “B” tumor, and in Figure 8D for a digitoxin-treated “A” tumor. For RNA isolation, this stained section was then used as a “stencil” to micropunch regions in the 300 micron sections that were rich in tumor cell islands. Libraries were prepared for transcription profiling of mRNAs on an Illumina platform. Approximately 17,000 mRNAs were identified and quantitated from each of the tumors. Supplemental Table 1 shows that 101 mRNAs out these *ca* 17,000 mRNAs were found to be different from control by at least 2-fold, and to be significantly different from control at the level of *P*, 2 tailed < 0.05. For convenience, we also include in this list an additional significant 81 mRNAs which only trend toward significance (*viz*, at least 2-fold change, but 0.05 < *P* < 0.10). Supplemental Table 2 lists 32 out of the 101 significantly affected mRNAs that are associated with cancer in the literature. Of these 32 mRNAs, 17 (*ca* 53%) are biomarkers for highly aggressive, poor outcome human CRPC.

### Gene Ontology Analysis of Digitoxin Targeted Genes

**Figure 9:**
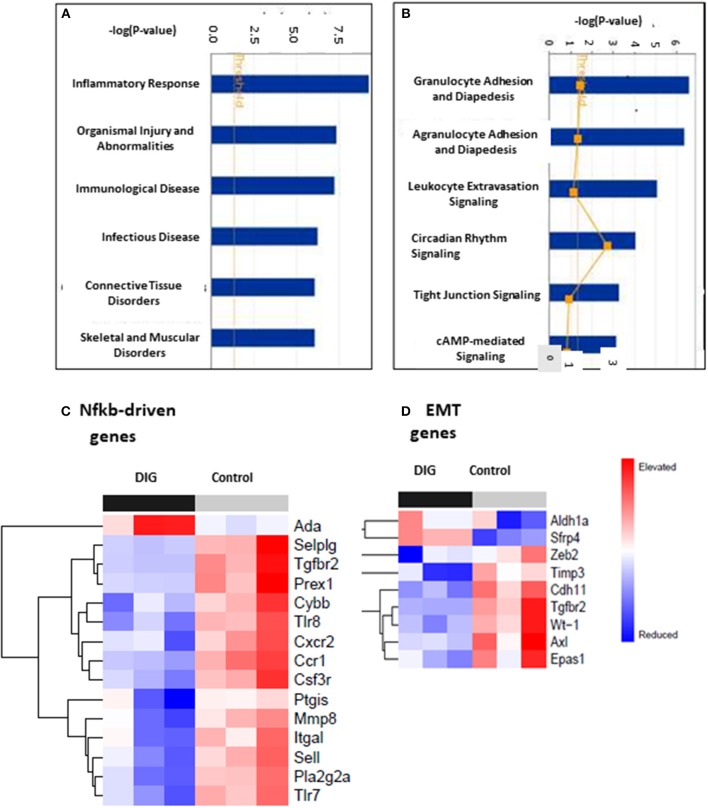
Gene ontology (GO) and Heat map analysis of digitoxin-dependent genes in stratified primary tumors. **(A)** Principal Gene Ontology (GO) analysis by IPA of digitoxin effects on transcription profiling of mRNA. The principal drug effects on mRNA and microRNA expression is on *Inflammatory Response* (#1, *P*, 2 tailed = *ca*. 10 ^−8^) and *Immunological Disease* (#3, *P*, 2tailed = *ca*. 10 ^−6^). Other significant and relevant categories include *Organismal Injury* and *Infectious Disease*. **(B)** Secondary Gene Ontology (GO) analysis by IPA of digitoxin effects on Inflammation on transcription profiling of mRNA. Within the category of the “inflammatory response” shown in Part A, the top three sub-GO categories refer to adhesion and diapedesis (movement of cells from blood to tissue) for granulocytes and agranulocytes, and for leukocyte extravasation signaling (all with *P*, 2 tailed < 1EXP-5). The fourth category is circadian rhythm signaling. **(C)** Effect of digitoxin on expression of Nfkb-driven mRNAs in stratified primary tumors. Among the top 15 genes in this category, 14, including Tgfbr2, are suppressed by treatment with digitoxin. **(D)** Effect of digitoxin on expression of Epithelia-to–Mesenchymal (EMT) mRNAs in stratified primary tumors. Of the 9 genes in this category, two are elevated (Aldh1a and Sfrp4) and 7 are reduced (Zeb4, Timp3, Cdh11, Tgfbr2, Wt-1, Axl, and Epas1).

To test for common targets of these digitoxin-dependent genes, we subjected the set of 101 (P, 2 tailed < 0.05) mRNAs to Gene Ontology (GO) analysis. Figure 9A shows that the principal effect of digitoxin is on *Inflammatory Response* (*P* = *ca*. 10 ^−8^). Figure 9B describes the results of a secondary gene ontology (GO) analysis of subcategories of *Inflammatory Response*. In ascending order of *P*-value, these include granulocyte adhesion and diapedesis (movement of neutrophils, eosinophils, and basophils from blood into tissue); agranulocyte adhesion and diapedesis (movement of lymphocytes and monocytes from blood into tissue); leukocyte extravasation signaling (a general term for all nucleated formed elements in the blood); circadian rhythm signaling; tight junction signaling (*viz*., epithelial-to-mesenchymal-transition, EMT); and cAMP-mediated signaling. Thus, this set of molecular characteristics identifies both cancer cells and their tumor microenvironment as gene expression targets for digitoxin.

### Digitoxin Suppresses Nfkb Signaling in Primary Tumors

To test whether the mechanism of digitoxin action in these CRPC tumors in the rat model might involve suppression of Nfkb-driven genes in primary tumors, we surveyed the set of all significantly affected mRNAs at the *P* < 0.05 level. Based on the presence of at least one functional Nfkb sequence, and supporting data from the literature, we identified 17 Nfkb-driven genes. Figure 9C shows a heatmap analysis of the top 15 genes. With the exception of Ada (adenosine deaminase), the remaining 14 are all suppressed by digitoxin. Furthermore, *Tgfbr2* expression is among those mRNAs that were significantly reduced by digitoxin in rat tumors. Thus, there appears to be a functional parallel, marked by digitoxin inhibition, between NFκB signaling to *TGFBR2* in human prostate PC-3 cancer cells, and Nfkb signaling to *Tgfbr2* in rat prostate cancer cells. In addition to *Tgfbr2*, increased expression of 9 of the reduced mRNAs in Figure 9C are also associated with enhanced metastasis and poor prognosis in human prostate cancer (see Supplemental Table 2). These are the human equivalents of Pla2g2a, Mmp8, Sell, Cxcr2, Csf3r, Ccr1, Selplg, Itgal, and Prex1.

### Digitoxin Suppresses mRNA Expression Associated With Epithelial-to Mesenchymal Transition (EMT)

To test for effects of chronic digitoxin therapy on EMT gene expression, we identified mRNAs associated with EMT in the entire significant dataset of 101 significantly modified mRNAs (see Supplementary Table 1). Figure 9D shows that of the 9 mRNAs in this category, 7 mRNAs are reduced by digitoxin treatment. In addition to Tgfbr2, these include Zeb2, Timp3, Cdh11, Tgfbr2, Wt-1 (Wilm's tumor suppressor gene 1), Axl (Axl receptor tyrosine kinase), and Epas1. Two of the 9 mRNAs are elevated by digitoxin, including Aldh1a and Sfrp4. We also tested the rat prostate tumors for digitoxin effects on Hspb1 and the *HSPB1/Rbfox2* ratio. The observed reductions, comparing 8 digitoxin-treated tumors and 8 control tumors, did not approach significance. However, in general, the ability of digitoxin to reduce EMT signaling observed in human prostate cancer PC3 cells would appear to have been replicated in this rat prostate cancer model.

### Digitoxin Elevates microRNA Expression Associated With Epithelial-to Mesenchymal Transition (EMT)

Finally, we also tested for possible associations between digitoxin-affected microRNAs (miRs) and EMT by screening 250 miRs in primary rat tumors for digitoxin effects. Table 3 identifies four miRs that are significantly affected by digitoxin. Three are elevated, including miR-204-5p/miR-211-5p, miR-448-3p, and miR-98/let 7a-5p, while miR-346 is reduced. The IPA-predicted interactions with digitoxin-affected mRNAs, and between the miRs themselves, are shown in Table 3B. In general, *elevation* of miRs is associated with *reduction* of target mRNAs. Thus, the IPA data in Table 3B, lower panel shows that the elevated miRs, coded in red, target mRNAs that are generally reduced in expression, and therefore coded in green. The connector between the miR let-7a-5p and miR-448-3p indicates that these two miRs control each other's expression. Two miRs, miR-346 and miR-448-3p, converge to suppress *ZEB2*, a classical EMT gene. The microRNA miR-204-5p targets TGFBR2, the hub receptor for TGFβ. The sequence relationships between microRNAs identified as either rat (rno) or mouse (mmu) and their human (hsa) counterparts are further delineated in Supplementary Table 3.

**Table 3:** Digitoxin effects on microRNA content in primary tumors.

**MicroRNA**	***p*-value**	**DDCt [Test, A–Control, B]**	**Fold-change (corrected to absolute values)**
**A**			
miR-346	0.034	+5.05	−33.1
miR-98/let7a-5p	0.019	−3.18	+9.06
miR-448-3p	0.026	−3.93	+15.24
miR-211-5p/miR-204-5p	0.040	−4.59	+24.08
**B**			
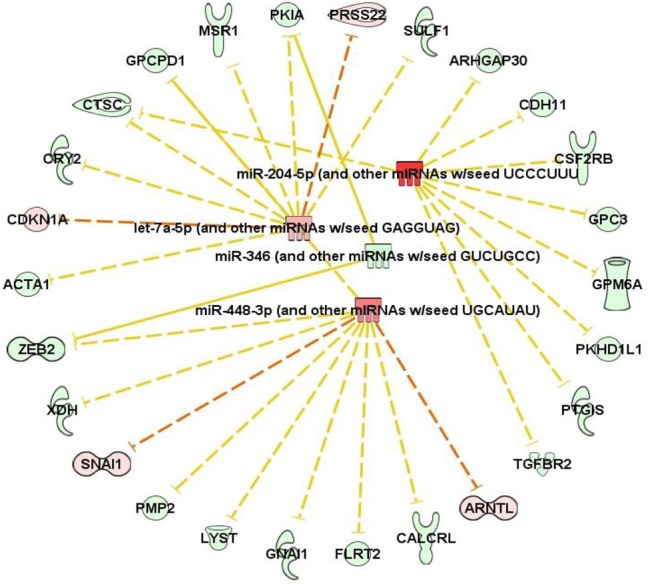			

*Panel A, DDCt (delta delta Ct) takes into account both the sample and a control. A negative or positive DDCt value corresponds to an increase or decrease, respectively, in fold change. A (+ digitoxin), n = 10; B (control), n = 11. Panel B, IPA analysis comparing predicted interactions of microRNAs with digitoxin-dependent mRNAs. Color Code: red (increased expression); green (decreased expression)*.

## Discussion

These data show that digitoxin suppresses signaling by both TNFα/NFκB and TGFβ/TGFBR2 pathways in castration resistant prostate cancer cells, both *in vitro* and *in vivo*, and that an important consequence is significant suppression of EMT, tumor progression and tumor recurrence. At the *in vitro* protein level in the human PC-3 cell system, digitoxin is not only able to suppress Vimentin expression while enhancing E-Cadherin, but is also able to block TGFBR2 expression. This inverse Vimentin and E-Cadherin response is a traditional signature for suppressing EMT. Furthermore, because TGFBR2 expression is driven by NFκB, and digitoxin blocks NFκB signaling, the suppression of TGFBR2 expression by digitoxin was expected and found. Consistently, digitoxin is also able to significantly suppress mRNA expression for both HSPB1, a biomarker for EMT in prostate cancer, and the HSPB1/RBFOX2 ratio. Finally, using RNA-seq to probe the *in vivo* rat prostate cancer system, digitoxin was found to suppress Nfkb signaling to multiple mRNAs, including Tgfbr2, as well as multiple EMT mRNAs. Unexpectedly, these include Wt-1 and Axl; both are important as drivers of human metastatic disease. These apparent parallels between digitoxin action in both systems therefore make it likely, as hypothesized, that digitoxin may work by breaking the collaborating process linking inflammation, TGFβ, and EMT.

In addition to digitoxin targeting mRNAs for inflammation and EMT, digitoxin also targets microRNAs which target the same functions. However, because of the small size of the targeting seed sequence, microRNAs can target the 3′ UTRs of multiple mRNAs, with binding energies proportional to the number of complementary bases linking the miR with many mRNAs. Thus, the functional significance of a potential interaction has to be assessed in a separate experiment. However, especially relevant to the present study is the fact that elevated miR-204 has been reported to form a functional complex with *TGFBR2* mRNA and to *reduce* TGFBR2 protein expression (41). In addition, let7 is reported to have tumor suppressor functions in human prostate cancer stem cells/progenitor cells (42). Furthermore, miR-346 is *elevated* in sera from both the TRAMP mouse model of prostate cancer, and from patients with CRPC (43). While a role for miR-448-3p in prostate cancer has not yet been reported, the seed sequence for miR-448-3p is predicted to target SNAI1, a key EMT-transcription factor (Table 3B, lower panel). Thus, these data demonstrate a close relationship between digitoxin action and target miRs with control of EMT functions, including TGFBR2.

In addition to directly targeting the collaborative mechanism linking NFκB and TGFβ to EMT, digitoxin treatment unexpectedly has systems-wide effects on multiple genes and processes associated with tumor progression and metastases. One possibility is that the systems-wide effects of digitoxin derive in large part from its ability to directly target the central control hubs for NFκB signaling and TGFβ signaling, which are thought to be collaboratively driving EMT. For example, the NFκB hub controls the *functional* expression of at least 784 NFκB-dependent downstream genes (44). It has also been reported that the TGFβ signaling pathway, forming another major biological hub at TGFBR2, itself affects the expression of as many as 1,235 genes (45). Therefore, by acting on the TNFα/NFκB hub (784 genes), and indirectly on the TGFβ hub through TGFBR2 (1,235 genes), this one drug alone is therefore *potentially* able to affect expression of at least 2019 genes, or approximately 10% of the *ca* 20,000 genes in the human genome. The common action on NFκB and TGFβ hubs may therefore provide a rationale for the polygenic action of this drug.

In conclusion, while the existence of a collaboration between NFκB and TGFβ to drive EMT and metastasis has been appreciated, this is the first report to show that chronically administered digitoxin breaks the collaboration, thus blocking tumor progression, EMT and ensuing metastatic CRPC disease. We cannot exclude the possibility that digitoxin, among the oldest and best studied drugs in the human pharmacopeia, could contribute to CRPC therapy in humans.

## Ethics Statement

All animal studies were approved by the University of Notre Dame Institutional Animal Care and Use Committee (IACUC), and were conducted in a facility accredited by the Association for Assessment and Accreditation of Laboratory Animal Care International (AAALAC).

## Author Contributions

BP contributed by conceiving the project, managing the research, and writing the paper. MAS contributed by managing the research and writing the paper. WW, PK, MES, and QY contributed by performing experiments. JS, OE, and SB contributed by analyzing the data. CD contributed by performing experiments, analyzing data, and writing the paper. RB contributed by performing experiments and writing the paper. HP contributed by designing experiments, managing the research, and writing the paper. XZ and MW performed bioinformatic analyses.

### Conflict of Interest Statement

BP is the sole invertor on an issued patent regarding digitoxin. The remaining authors declare that the research was conducted in the absence of any commercial or financial relationships that could be construed as a potential conflict of interest.
