# Supplementary material for: Digitoxin Inhibits Epithelial-to-Mesenchymal-Transition in Hereditary Castration Resistant Prostate Cancer
Source: Front Oncol. 2019 Aug 2;9:630. doi: 10.3389/fonc.2019.00630 (PMC6687970; doi:10.3389/fonc.2019.00630)
Supplement: Supplementary file 1 [file Table_1.DOCX]

**Supplementary Table 1. Digitoxin effect on Transcription profiling of primary tumors from LW rats bearing CRPC tumors, FPKM^#^**

(# FPKM is Fragments per Kilobase of Gene per Million Reads.)

| **Gene, mRNA** | **locus** | **wi/out Digitoxin, FPKM^#^** | **+ Digitoxin, FPKM^#^** | **log2(fold_change)** | **p_value** |
| --- | --- | --- | --- | --- | --- |
| Pla2g2a | chr5:161024072-161026650 | 614.469 | 54.856 | -3.48562 | 5.00E-05 |
| Prph | chrX:115247358-115251138 | 34.3597 | 2.42025 | -3.82749 | 5.00E-05 |
| LOC360228 | chr10:70671086-70676956 | 252.048 | 31.2702 | -3.01084 | 0.0003 |
| Mmp8 | chr8:5771723-5780576 | 33.9721 | 5.68813 | -2.57832 | 0.0003 |
| Sell | chr13:87249242-87267197 | 80.6358 | 23.0961 | -1.80377 | 0.0004 |
| Cyp2a3 | chr1:84869279-84876600 | 40.9358 | 7.23067 | -2.50116 | 0.0013 |
| Csf3 | chr10:86413102-86415477 | 19.9512 | 62.7056 | 1.65212 | 0.0019 |
| Steap4 | chr4:23040107-23062780 | 52.5064 | 19.0123 | -1.46556 | 0.00265 |
| Cxcr2 | chr9:81193441-81199814 | 33.1252 | 10.4747 | -1.66102 | 0.00285 |
| Rps27a | chr14:113632953-113634784 | 2.09349 | 18.4921 | 3.14293 | 0.00315 |
| Csf3r | chr5:147793515-147820326 | 30.5524 | 11.0466 | -1.46769 | 0.0032 |
| Dbp | chr1:102766761-102771674 | 72.5397 | 31.9925 | -1.18104 | 0.0032 |
| Slurp1 | chr7:115867043-115868460 | 41.6481 | 4.8645 | -3.09789 | 0.0033 |
| Reg3g | chr4:174231309-174233865 | 19.0564 | 0.452045 | -5.39767 | 0.0035 |
| Upk1b | chr11:67555643-67586433 | 44.2316 | 15.9906 | -1.46786 | 0.00405 |
| Mcpt2 | chr15:38920068-38922403 | 41.7687 | 8.73742 | -2.25714 | 0.0045 |
| Mpeg1 | chr1:235886915-235889176 | 29.7253 | 11.4492 | -1.37644 | 0.0055 |
| Gda | chr1:246270845-246345967 | 33.7402 | 11.7886 | -1.51708 | 0.0057 |
| Pkhd1l1 | chr7:83387128-83563775 | 3.35089 | 0.958649 | -1.80547 | 0.0058 |
| Timp3 | chr7:23553830-23958150 | 72.6299 | 28.9819 | -1.32541 | 0.0058 |
| Spon2 | chr14:83504581-83510376 | 4.93469 | 16.248 | 1.71923 | 0.00595 |
| Aldh1a2 | chr8:75363284-75442554 | 23.2847 | 8.17268 | -1.5105 | 0.00605 |
| Pmp2 | chr2:113582540-113586456 | 5.51307 | 1.09638 | -2.33011 | 0.0061 |
| C1s | chr4:224162095-224174100 | 52.7165 | 25.7977 | -1.03101 | 0.0072 |
| Per2 | chr9:98231203-98273371 | 8.61326 | 3.67305 | -1.22958 | 0.0078 |
| Ccr1 | chr8:132147925-132153481 | 20.7715 | 7.92142 | -1.39077 | 0.0082 |
| Hpd | chr12:40729051-40740668 | 7.97637 | 2.0089 | -1.98933 | 0.0089 |
| Gpm6a | chr16:39144852-39253973 | 25.0131 | 10.1381 | -1.30289 | 0.00925 |
| Selplg | chr12:50359788-50361080 | 39.1112 | 16.3355 | -1.25957 | 0.0094 |
| Cd53 | chr2:228956378-229004197 | 141.703 | 71.9864 | -0.977079 | 0.0106 |
| Cybb | chrX:15359404-15391317 | 27.6781 | 11.4349 | -1.27531 | 0.01065 |
| LOC689064 | chr1:175098754-175100120 | 60.2107 | 17.0868 | -1.81714 | 0.0114 |
| Hr | chr15:55969002-55984791 | 8.77947 | 18.8871 | 1.1052 | 0.01145 |
| Reg3a | chr4:174183720-174186487 | 1.58181 | 0 | undefined | 0.012 |
| Npas2 | chr9:45590020-45769792 | 1.05193 | 3.18512 | 1.5983 | 0.0127 |
| Flrt2 | chr6:128739107-128828633 | 2.45511 | 0.594696 | -2.04556 | 0.0155 |
| Sfrp4 | chr17:56095165-56105248 | 7.31764 | 21.5232 | 1.55644 | 0.01705 |
| Cldn15 | chr12:24751107-24758228 | 12.2001 | 3.09799 | -1.97749 | 0.01775 |
| St6galnac1 | chr10:105374444-105384844 | 1.08853 | 0.155784 | -2.80477 | 0.0179 |
| Nptxr | chr7:121002636-121020713 | 9.85699 | 21.0656 | 1.09567 | 0.0186 |
| Per3 | chr5:171665220-171700125 | 6.6211 | 2.73416 | -1.27597 | 0.0195 |
| Calcrl | chr3:78271812-78366301 | 18.5156 | 6.82796 | -1.43921 | 0.0197 |
| Acta1 | chr19:67389898-67392911 | 2.22194 | 0.343371 | -2.69398 | 0.02065 |
| Oas1k | chr12:43152976-43165724 | 35.9245 | 16.6835 | -1.10655 | 0.02115 |
| Slc7a9 | chr1:92843092-92865757 | 2.02648 | 0.257601 | -2.97576 | 0.02125 |
| Ccdc80 | chr11:64747814-64781404 | 69.273 | 38.4561 | -0.849079 | 0.02145 |
| Ptgis | chr3:170109316-170143882 | 32.6926 | 14.2963 | -1.19332 | 0.0218 |
| Mab21l3 | chr2:223666826-223689474 | 4.41281 | 10.2371 | 1.21404 | 0.02225 |
| Axl | chr1:83812117-83840542 | 28.1887 | 14.1872 | -0.990524 | 0.02305 |
| Ly6al | chr7:116449847-116453387 | 20.5895 | 6.81855 | -1.59437 | 0.02315 |
| Gng8 | chr1:80067666-80069629 | 13.5679 | 36.9301 | 1.4446 | 0.0239 |
| Fxyd4 | chr4:215913321-215917183 | 39.09 | 14.493 | -1.43145 | 0.02435 |
| Cmah | chr17:44449933-44506535 | 4.67981 | 1.26418 | -1.88825 | 0.0245 |
| Slpil2 | chr3:166921183-166922634 | 108.264 | 43.2013 | -1.32541 | 0.0246 |
| Csf2rb | chr7:119544872-119558539 | 19.6886 | 9.876 | -0.995364 | 0.02525 |
| Xdh | chr6:34996982-35059195 | 51.7311 | 27.1885 | -0.928035 | 0.02555 |
| Actg1 | chr10:109113704-109116103 | 23.8538 | 80.7038 | 1.75842 | 0.02595 |
| Fam65b | chr17:44192187-44289647 | 3.52408 | 1.11437 | -1.66102 | 0.02595 |
| Slc17a3 | chr17:45373768-45398194 | 0.174458 | 1.10465 | 2.66264 | 0.0266 |
| Pik3ap1 | chr1:267983940-268093777 | 9.11847 | 3.80994 | -1.25902 | 0.0267 |
| Bpifb1 | chr3:156046294-156078740 | 17.9301 | 7.30668 | -1.2951 | 0.02795 |
| Crlf1 | chr16:20530520-20541796 | 28.2198 | 51.4825 | 0.867377 | 0.02855 |
| Mt1a | chr19:11277132-11278149 | 79.4713 | 168.766 | 1.08652 | 0.0292 |
| Arntl | chr1:185007567-185105413 | 6.27606 | 12.4927 | 0.993158 | 0.02945 |
| Rpl13 | chr19:66622823-66625371 | 49.3913 | 93.6795 | 0.923477 | 0.03035 |
| Tgfbr2 | chr8:123585764-123671209 | 34.0495 | 16.4172 | -1.05242 | 0.03065 |
| Wt1 | chr3:101756492-101802600 | 5.07066 | 1.9584 | -1.3725 | 0.03085 |
| Scgb3a1 | chr10:34905079-34906387 | 34.0017 | 4.27781 | -2.99066 | 0.03115 |
| Oas3 | chr12:43176425-43194139 | 2.81759 | 0.74427 | -1.92056 | 0.0323 |
| Myl4 | chr10:92390124-92398028 | 4.71095 | 1.01936 | -2.20835 | 0.0328 |
| LOC681325 | chrX:80538522-80542136 | 12.251 | 5.74951 | -1.09139 | 0.0331 |
| Cxcl13 | chr14:15193474-15198511 | 28.3071 | 12.8149 | -1.14334 | 0.03325 |
| Itgal | chr1:205742368-205780281 | 24.1468 | 12.6442 | -0.933352 | 0.03455 |
| Prss22 | chr10:13186015-13190726 | 153.877 | 332.803 | 1.11289 | 0.03555 |
| Gata4 | chr15:50151498-50197708 | 2.36485 | 0.600926 | -1.97649 | 0.0357 |
| Efcab1 | chr11:93274701-93287932 | 1.84865 | 0.202691 | -3.18912 | 0.0365 |
| Mcpt8l2 | chr15:38512230-38515184 | 5.9704 | 1.22386 | -2.28639 | 0.03675 |
| Rps14 | chr18:55276796-55281581 | 0.587599 | 5.59923 | 3.25232 | 0.03675 |
| Clec4a3 | chr4:222933505-222943219 | 38.1228 | 18.0975 | -1.07487 | 0.0379 |
| Bpifa1 | chr3:156002439-156008183 | 2.77698 | 0.0167989 | -7.36901 | 0.03845 |
| Nt5e | chr8:95464590-95508299 | 10.7688 | 5.56627 | -0.952079 | 0.04045 |
| Klf2 | chr16:19084195-19086146 | 56.6962 | 27.356 | -1.0514 | 0.0406 |
| Prex1 | chr3:169494330-169575981 | 7.70885 | 3.85609 | -0.999376 | 0.04125 |
| Cald1 | chr4:61952323-62021822 | 20.3157 | 10.8054 | -0.910841 | 0.0414 |
| Padi1 | chr5:163045277-163077738 | 4.45752 | 8.84532 | 0.988674 | 0.04175 |
| LOC100134871 | chr1:175127945-175136083 | 3.54451 | 0 | undefined | 0.04345 |
| Cdh11 | chr19:2374107-2531571 | 4.92238 | 2.51664 | -0.967856 | 0.04505 |
| Npl | chr13:75903276-75946056 | 12.3612 | 6.48197 | -0.931313 | 0.0452 |
| Ada | chr3:166306000-166330108 | 26.9573 | 50.4248 | 0.903458 | 0.04545 |
| Man1a1 | chr20:37015822-37203609 | 12.4967 | 6.67367 | -0.904999 | 0.04565 |
| Fcnb | chr3:11974690-11983068 | 21.8358 | 9.78164 | -1.15855 | 0.0462 |
| Gnai1 | chr4:13388069-13470279 | 7.15682 | 2.66548 | -1.42492 | 0.0473 |
| Cyp2f4 | chr1:85146273-85158525 | 19.5872 | 7.98618 | -1.29433 | 0.04795 |
| C1r | chr4:224144563-224155321 | 73.0442 | 44.3463 | -0.719958 | 0.0501 |
| Cpa3 | chr2:124745510-124777636 | 16.4873 | 6.96069 | -1.24405 | 0.0501 |
| Lrat | chr2:201310923-201319986 | 3.08128 | 1.40276 | -1.13526 | 0.05025 |
| Cd38 | chr14:71746254-71785712 | 20.4834 | 11.5413 | -0.827646 | 0.0517 |
| Dab2 | chr2:75489437-75510929 | 53.6134 | 32.023 | -0.743487 | 0.052 |
| Idh1 | chr9:70668352-70689596 | 42.8469 | 25.2822 | -0.761068 | 0.0534 |
| Atg9b | chr4:7325732-7332393 | 9.06665 | 16.9616 | 0.903636 | 0.0542 |
| Cebpa | chr1:92493856-92495245 | 20.9636 | 10.3955 | -1.01192 | 0.0543 |
| Rasa3 | chr16:80808491-80922641 | 16.1693 | 8.84374 | -0.870527 | 0.05505 |
| Ptrf | chr10:88659594-88671569 | 71.5213 | 40.5167 | -0.819856 | 0.0554 |
| 1-Mar | chr16:24885331-25407999 | 4.82656 | 1.29322 | -1.90003 | 0.05575 |
| Bicc1 | chr20:20939897-21092147 | 3.52083 | 1.49421 | -1.23653 | 0.0563 |
| Slc6a6 | chr4:188176237-188245992 | 10.3143 | 5.06303 | -1.02657 | 0.05755 |
| H1foo | chr4:211128368-211133490 | 1.02158 | 3.65356 | 1.83851 | 0.0578 |
| LOC501738 | chr10:103127533-103137523 | 2.63068 | 0.497644 | -2.40225 | 0.05825 |
| Arhgap30 | chr13:94402578-94423288 | 20.5572 | 11.9273 | -0.785379 | 0.05975 |
| B4galnt4 | chr1:220789703-220800586 | 9.66162 | 17.3208 | 0.842171 | 0.0601 |
| Gapdh | chr4:224693579-224697452 | 69.2952 | 123.395 | 0.832458 | 0.0605 |
| Pde1b | chr7:142899028-142924626 | 2.63447 | 0.680667 | -1.95249 | 0.0608 |
| Tlr7 | chrX:28883040-28908313 | 4.88326 | 2.29048 | -1.09219 | 0.06155 |
| Ctsc | chr1:158231533-158263124 | 125.843 | 78.6489 | -0.678127 | 0.06175 |
| Rasgrp4 | chr1:89253161-89269484 | 6.73426 | 2.89409 | -1.21841 | 0.0619 |
| Akap12 | chr1:42162162-42252615 | 11.7034 | 19.9011 | 0.76592 | 0.06195 |
| Lilrb3l | chr1:62834636-62841318 | 5.77654 | 2.31186 | -1.32115 | 0.0628 |
| Clec4e | chr4:223287985-223293143 | 44.7989 | 23.4072 | -0.936512 | 0.0629 |
| Has1 | chr1:60640269-60652067 | 2.77307 | 1.01406 | -1.45135 | 0.06405 |
| Chst4 | chr19:52455334-52463987 | 3.18206 | 0.821904 | -1.95292 | 0.06525 |
| Clec5a | chr4:133598268-133607250 | 11.5367 | 5.51353 | -1.06519 | 0.06625 |
| Cfh | chr13:61997443-62094826 | 48.4773 | 29.2511 | -0.728816 | 0.06645 |
| Suv420h2 | chr1:75861336-75868943 | 10.8603 | 18.5676 | 0.773726 | 0.06875 |
| Epas1 | chr6:20299602-20377837 | 66.1269 | 31.9133 | -1.05108 | 0.06945 |
| Cdkn1a | chr20:8592436-8602879 | 55.1791 | 89.566 | 0.698831 | 0.0698 |
| Msr1 | chr16:56517028-56582240 | 31.6789 | 18.3335 | -0.789036 | 0.07 |
| Ccdc152 | chr2:72138103-72170595 | 1.7647 | 2.41583 | 0.453098 | 0.07005 |
| Sla | chr7:107399164-107602400 | 20.1076 | 10.8424 | -0.891053 | 0.07005 |
| Nlrp3 | chr10:45649990-45674234 | 6.77354 | 3.66448 | -0.886301 | 0.07125 |
| P2ry13 | chr2:168848654-168849665 | 4.38179 | 1.30147 | -1.75138 | 0.0721 |
| Fcrls | chr2:205998854-206009505 | 37.4301 | 22.5789 | -0.729225 | 0.07235 |
| Angptl4 | chr7:18805413-18811643 | 14.52 | 27.3446 | 0.91322 | 0.0724 |
| Hmgcs2 | chr2:219928568-219954863 | 3.17322 | 7.29366 | 1.20069 | 0.0732 |
| F2rl2 | chr2:45371779-45376928 | 18.3891 | 9.81921 | -0.905174 | 0.07355 |
| Zc3h12a | chr5:146878970-146887760 | 12.7204 | 21.6071 | 0.764366 | 0.07355 |
| Cyp26b1 | chr4:180848037-180864857 | 2.82183 | 5.33141 | 0.917884 | 0.0739 |
| Cyfip2 | chr10:31097978-31235818 | 7.59073 | 4.3145 | -0.815046 | 0.07405 |
| Mertk | chr3:128671361-128777078 | 4.61139 | 2.18025 | -1.08071 | 0.07535 |
| Iapp | chr4:240724562-240729478 | 13.203 | 1.65748 | -2.9938 | 0.07885 |
| Ccdc64 | chr12:48413928-48501896 | 9.19774 | 4.64861 | -0.98448 | 0.07905 |
| RGD1560455 | chrX:78457231-78482003 | 2.72497 | 1.14212 | -1.25453 | 0.07975 |
| Cxcl5 | chr14:18770998-18771910 | 425.324 | 760.883 | 0.839113 | 0.0804 |
| Zfp703 | chr16:68911229-68915401 | 22.905 | 35.8976 | 0.648221 | 0.0807 |
| Fmo3 | chr13:85732370-85750452 | 11.5904 | 5.51329 | -1.07195 | 0.08075 |
| Tlr8 | chrX:28932433-28955156 | 3.31198 | 1.4535 | -1.18816 | 0.08075 |
| RGD1304884 | chr19:64452678-64469186 | 9.53492 | 5.13999 | -0.891456 | 0.08165 |
| Mospd2 | chrX:31520481-31562128 | 27.8769 | 17.7014 | -0.655205 | 0.0823 |
| Nr1d2 | chr15:12801476-12816719 | 19.2038 | 11.1709 | -0.781642 | 0.0823 |
| Crocc | chr5:163252705-163295065 | 7.57332 | 12.6763 | 0.743136 | 0.0835 |
| Sulf1 | chr5:10835825-11022624 | 12.4535 | 7.40702 | -0.749584 | 0.08355 |
| Aoah | chr17:57368444-57604881 | 6.60063 | 3.01885 | -1.12861 | 0.08415 |
| Themis2 | chr5:154639450-154653218 | 7.99762 | 3.88001 | -1.04351 | 0.0847 |
| Il10ra | chr8:48186629-48200159 | 7.16521 | 3.30083 | -1.11818 | 0.08495 |
| Zeb2 | chr3:35063908-35185403 | 5.38798 | 2.80848 | -0.939955 | 0.0862 |
| Clec4a2 | chr4:222810974-222885994 | 13.819 | 6.22434 | -1.15066 | 0.0863 |
| Wee1 | chr1:181767889-181786293 | 15.4267 | 9.54442 | -0.692699 | 0.08795 |
| Sdpr | chr9:54940737-54952755 | 97.4391 | 55.0986 | -0.822485 | 0.0884 |
| Mybpc2 | chr1:101572090-101595570 | 1.19463 | 0.391204 | -1.61057 | 0.08845 |
| Creg1 | chr13:88852215-88864665 | 56.1693 | 35.1876 | -0.674714 | 0.0897 |
| Lilrc2 | chr1:69994292-69998713 | 14.2964 | 7.01936 | -1.02624 | 0.08995 |
| Rplp2 | chr1:221345391-221347663 | 19.6982 | 52.2923 | 1.40853 | 0.09015 |
| Vil1 | chr9:81454350-81482172 | 2.23627 | 4.35804 | 0.962585 | 0.091 |
| Asah1 | chr16:53712314-53743717 | 133.026 | 87.084 | -0.611231 | 0.09105 |
| Lpl | chr16:22432083-22455884 | 45.6914 | 27.2105 | -0.747763 | 0.0913 |
| Snx10 | chr4:145977453-146040126 | 14.2565 | 7.64629 | -0.898786 | 0.0923 |
| Rassf2 | chr3:131075322-131109910 | 4.74564 | 2.45699 | -0.949713 | 0.093 |
| Ikzf1 | chr14:91567473-91652247 | 3.20662 | 1.26208 | -1.34525 | 0.0936 |
| Sepw1 | chr1:79068031-79073104 | 0.394596 | 2.52675 | 2.67884 | 0.09525 |
| Lyst | chr17:91983425-92183054 | 3.50961 | 2.06998 | -0.761696 | 0.09565 |
| Tspan12 | chr4:48641400-48715044 | 31.3941 | 18.8148 | -0.738623 | 0.09585 |
| Gpcpd1 | chr3:131661587-131706004 | 24.7781 | 15.2703 | -0.698338 | 0.09635 |
| Itgbl1 | chr15:113495870-113759208 | 5.37122 | 2.63622 | -1.02678 | 0.0964 |
| Cry2 | chr3:88020047-88048168 | 3.9033 | 1.78126 | -1.1318 | 0.09665 |
| Pik3r5 | chr10:54802424-54822854 | 5.02525 | 2.47902 | -1.01942 | 0.09745 |
| Samhd1 | chr3:158468643-158504183 | 12.3916 | 7.02423 | -0.818947 | 0.09755 |
| Clca1 | chr2:269425676-269451501 | 0.342959 | 1.26562 | 1.88373 | 0.09835 |
| Slc17a4 | chr17:45314639-45325707 | 3.92752 | 7.46474 | 0.926474 | 0.0986 |
| Cd48 | chr13:94737446-94760975 | 147.641 | 91.1718 | -0.695434 | 0.0988 |
| Cd4 | chr4:224400829-224425954 | 13.5777 | 7.65504 | -0.826757 | 0.09965 |
| Tef | chr7:123019281-123034386 | 44.5525 | 26.3453 | -0.757959 | 0.1004 |
| Lrrc32 | chr1:169649229-169661088 | 6.28708 | 10.3752 | 0.722682 | 0.101 |
| Fibin | chr3:108150071-108152343 | 12.9752 | 21.0279 | 0.696554 | 0.1016 |
| Selm | chr14:84440339-84442922 | 68.1645 | 108.151 | 0.665955 | 0.10235 |
| Fgr | chr5:154839095-154866417 | 56.0057 | 35.9262 | -0.640539 | 0.1025 |
| Fbn1 | chr3:124095125-124289500 | 7.03139 | 4.4455 | -0.661463 | 0.10385 |
| Mier2 | chr7:13203326-13219032 | 6.79167 | 11.5424 | 0.765101 | 0.10445 |
| Ppp2r2c | chr14:78644678-78727963 | 1.25606 | 0.167638 | -2.90549 | 0.1052 |
| Snai1 | chr3:170425897-170430384 | 15.8529 | 27.3019 | 0.78426 | 0.106 |
| Gpc3 | chrX:139625382-139993328 | 98.5163 | 55.7194 | -0.822182 | 0.10615 |
| Emilin1 | chr6:36636869-36644587 | 12.0714 | 20.0196 | 0.729814 | 0.1076 |
| Ccl2 | chr10:69047090-69048889 | 72.0815 | 44.0471 | -0.710584 | 0.1085 |
| Samsn1 | chr11:17910039-17960596 | 17.812 | 9.80124 | -0.861817 | 0.10895 |
| Alcam | chr11:53964951-54165636 | 59.0238 | 35.3071 | -0.741338 | 0.10945 |
| Cox4i2 | chr3:154637662-154648535 | 11.9899 | 23.6294 | 0.97876 | 0.10955 |
| Rgs18 | chr13:66552685-66578436 | 5.39682 | 2.15959 | -1.32135 | 0.1097 |
| RGD1564093 | chr19:37118342-37121759 | 52.8866 | 85.6368 | 0.695329 | 0.11045 |
| Fosl1 | chr1:227755886-227764393 | 80.8962 | 132.634 | 0.713303 | 0.11055 |
| Pkia | chr2:116338939-116411705 | 2.34407 | 0.844281 | -1.47322 | 0.11075 |
| Dkc1 | chr1:151564353-151578635 | 56.58 | 85.544 | 0.596374 | 0.111 |
| Rbp4 | chr1:264286997-264294200 | 18.2973 | 8.86724 | -1.04507 | 0.111 |
