# Supplementary material for: Digitoxin Inhibits Epithelial-to-Mesenchymal-Transition in Hereditary Castration Resistant Prostate Cancer
Source: Front Oncol. 2019 Aug 2;9:630. doi: 10.3389/fonc.2019.00630 (PMC6687970; doi:10.3389/fonc.2019.00630)
Supplement: Supplementary file 2 [file Table_2.DOCX]

**Supplementary Table 2**

**Supplemental Table 2. Digitoxin reduces mRNA expression in PAIII primary tumors that are associated with tumor progression and metastases, selected from 101 mRNAs (p < 0.05).**

| **GENE, mRNA** | **Drug Effect, FPKM ***  **(p value)** | | **Action**  **(prostate cancer)** | **Reference** |
| --- | --- | --- | --- | --- |
| **Pla2g2a**  **(Phospholipase A2, group 2a)** | **614 → 55**  **(p = 5E-05)** | | **Biomarker for tumor progression, poor prognosis in prostate cancer** | **(**[**1**](#_ENREF_1)**) (**[**2**](#_ENREF_2)**) (**[**3**](#_ENREF_3)**)** |
| **Mmp8**  **(Matrix metalloprotease 8)** | **33.9 → 5.7**  **(p = 0.0003)** | | **Induces IL-6,IL-8 in cancer cells** | **(**[**4**](#_ENREF_4)**)** |
|  | | | **Inactivates beta-1 integrins in prostate cancer cells** | **(**[**5**](#_ENREF_5)**)** |
| **Sell**  **(Selectin L; cell adhesion)** | | **80.8 → 23.1**  **(p = 0.0004)** | **Mediates cancer cell adhesion, cytokine release, and metastasis** | **(**[**6**](#_ENREF_6)**)** |
| **Steap4 (Six transmembrane epithelial antigen of prostate 4)** | | **52.5→19.0**  **(p = 0.0026)** | **Overexpressed in human prostate cancer** | **(**[**7**](#_ENREF_7)**)** |
| **Cxcr2**  **(Receptor for CINC1 [ “IL-8” equivalent in rat]** | | **33.1 →10.5**  **(p = 0.003)** | **Recruits tumor-promoting PMNL’s** | **(**[**8**](#_ENREF_8)**) (**[**8**](#_ENREF_8)**)** |
|  | |  | **Promotes tumor growth, angiogenesis, metastasis** | **(**[**9**](#_ENREF_9)**)** |
| **Csf3r**  **(Colony stimulating factor 3 receptor)** | | **30.6→11.0**  **(p = 0.003)** | **Highest levels in prostate tumors with highest Gleason scores** | **(**[**10**](#_ENREF_10)**) (**[**11**](#_ENREF_11)**)** |
| **Aldh1a2 (Aldehyde dehydrogenase-1** | | **23.3 → 8.2**  **(p = 0.006)** | **EMT marker; elevated in highly aggressive, poor prognosis tumors** | **(**[**12**](#_ENREF_12)**) (**[**13**](#_ENREF_13)**) (**[**14**](#_ENREF_14)**) (**[**15**](#_ENREF_15)**)** |
| **Csf3 (Granulocyte Colony Stimulating Factor)** | | **19.9 → 10.5**  **(p = 0.002)** | **Promotes prostate cancer cell metastasis to bone** | **(**[**16**](#_ENREF_16)**)** |
| **Ccr1 (chemokine(CC-motif) receptor 1** | | **20.8 → 7.9**  **(p = 0.008)** | **Stimulates cancer cell invasion;** | **(**[**17**](#_ENREF_17)**) (**[**18**](#_ENREF_18)**)** |
| **Selplg (Selectin P)** | | **39.1 →16.3**  **(p = 0.009)** | **Promotes metastasis;** | **(**[**19**](#_ENREF_19)**) (**[**20**](#_ENREF_20)**) (**[**21**](#_ENREF_21)**)** |
| **CD53 (tetraspanin 25)** | | **141.7 → 72.0**  **(p = 0.01)** | **Metastasis susceptibility gene** | **(**[**22**](#_ENREF_22)**)** |
| **Calcr1 (calcitonin)** | | **18.5 →6.8**  **(p = 0.02)** | **Increases angiogenic activity in PC3 cells** | **(**[**23**](#_ENREF_23)**)** |
| **Ccdc80 (coiled-coli doman containing 80)** | | **69.3 → 38.6**  **(p = 0.02)** | **Tumor suppressor gene** | **(**[**24**](#_ENREF_24)**)** |
| **Ptgis (prostaglandin 12 synthase)** | | **32.7 → 14.3**  **(p = 0.02)** | **Correlates with poor cancer prognosis; induces VEGF synthesis** | **(**[**25**](#_ENREF_25)**) (**[**26**](#_ENREF_26)**)** |
| **Fxyd4 (CHIF; FXYD domain containing ion transport regulator 4)** | | **39.1 → 14.5**  **(p = 0.02)** | **Binds to tissue-specific subunits of NaKATPase (alternative digitoxin target?)** | **(**[**27**](#_ENREF_27)**)** |
| **Cmah ( Cytidine monophosphate-N acetylneuraminic acid hydrolase** | | **4.7 → 1.3**  **(p = 0.02)** | **Stem cell marker** | **(**[**28**](#_ENREF_28)**)** |
| **Axl (“anexelekto” receptor tyrosine kinase** | | **28.2 → 14.2**  **(p = 0.02)** | **Drives proliferation, migration, invasion in prostate tumor cells *in vitro* and tumors *in vivo*** | **(**[**29**](#_ENREF_29)**)** |
| **Csf2rb (Colony stimulating factor 2 receptor)** | | **19.7 → 9.9**  **(p = 0.03)** | **Elevation is a biomarker for poor prognosis in some tumors** | **(**[**30**](#_ENREF_30)**)** |
| **Fam65b (Family with sequence similarity 65, member B)** | | **3.5 →1.1**  **(p = 0.03)** | **Marker for cancer stem cells in PC3 CRPC cells** | **(**[**31**](#_ENREF_31)**)** |
| **Pik3ap1 (BCAP, phosphoinositide-3 kinase adaptor protein 1)** | | **9.1 → 3.8**  **(p = 0.03)** | **Mediates PIK3 activation; PI3K pathway gene associated with risk of prostate cancer** | **(**[**32**](#_ENREF_32)**) (**[**33**](#_ENREF_33)**)** |
| **Wt1 (Wilms’tumor gene)** | | **5.1 →2.0**  **(p = 0.03)** | **Biomarker for prostate cancer progression; elevated in high Gleason score prostate cancer** | **(**[**34**](#_ENREF_34)**) (**[**35**](#_ENREF_35)**)** |
| **Cxcl-13 (C-X-C motif cytokine 13)** | | **28.3 → 12.8**  **(p = 0.03)** | **Supports prostate cancer cell invasion, mediated by PI3K signaling** | **(**[**36**](#_ENREF_36)**)** |
| **Tgfbr2 (TGF-β type II receptor)** | | **34.0 → 16.4**  **(p = 0.03)** | **Contributes to prostate cancer initiation, progression, invasion** | **(**[**37**](#_ENREF_37)**)** |
| **Itgal (integrin alpha L)** | | **24.1 → 12.6**  **(p = 0.03)** | **Biomarker for high risk CRPC** | **(**[**38**](#_ENREF_38)**)** |
| **Rps14 (Ribosomal protein S 14)** | | **0.6→5.6**  **(p = 0.04)** | **Activates a miR-dependent reduction in cMYC mRNA** | **(**[**39**](#_ENREF_39)**)** |
| **Prex1 (PIP3-dependent Rac exchanger 1)** | | **7.7 → 3.9**  **(p = 0.04)** | **Up-regulation promotes prostate cancer metastasis** | **(**[**40**](#_ENREF_40)**)** |
| **Cald1(caldesmon1)** | | **20.3 → 10.8**  **(p = 0.04)** | **Promotes cancer cell migration and invasion** | **(**[**41**](#_ENREF_41)**)** |
| **Padi1 (peptidyl arginine deiminase)** | | **4.5 → 8.8.**  **(p =0.04)** | **Citrulination of CXCL8 (IL-8) reduces activity** | **(**[**42**](#_ENREF_42)**)** |
| **Cdh11 (cadherin 11)** | | **4.9 → 2.5**  **(p = 0.045)** | **mediates metastasis of prostate cancer cells to bone** | **(**[**43**](#_ENREF_43)**)** |
| **Dab2(mitogen-responsive phosphoprotein, homolog 2)** | | **53.6→32.0**  **(p = 0.05)** | **Required for TGFβ-induced VEGF expression in cancer cells** | **(**[**44**](#_ENREF_44)**)** |
| **Atg9b (autophagy related 9B)** | | **9.1→ 17.0**  **(p= 0.05)** | **Activates autophagy; induces cancer cell senescence** | **(**[**45**](#_ENREF_45)**) (**[**46**](#_ENREF_46)**)** |
| **Cebpa (C/EBP, CCAAT/enhancer binding protein)** | | **21.0 → 10.4**  **(p = 0.05)** | **Increased in high Gleason score prostate cancer** | **(**[**47**](#_ENREF_47)**)** |
| **Ptrf (RNA polymerase 1 and transcript release factor** | | **71.5 → 40.5**  **p = 0.055** | **Ribosome synthesis; decreases migration of PC3 cells; mediates angiogenesis in prostate cancer** | **(**[**48**](#_ENREF_48)**) (**[**49**](#_ENREF_49)**)** |
| **Epas1 (Hif2a, endothelial PAS domain protein 1)** | | **66.1 →32.0**  **(p = 0.07)** | **VEGF induction; High levels associated with poor outcomes in different cancers** | **(**[**50**](#_ENREF_50)**) (**[**51**](#_ENREF_51)**)** |

**References for Supplementary Table 2**

1. Oleksowicz L, Liu Y, Bracken RB, Gaitonde K, Burke B, Succop P, et al. Secretory phospholipase A2-IIa is a target gene of the HER/HER2-elicited pathway and a potential plasma biomarker for poor prognosis of prostate cancer. The Prostate. 2012;72(10):1140-9.

2. Dong Z, Liu Y, Scott KF, Levin L, Gaitonde K, Bracken RB, et al. Secretory phospholipase A2-IIa is involved in prostate cancer progression and may potentially serve as a biomarker for prostate cancer. Carcinogenesis. 2010;31(11):1948-55.

3. Menschikowski M, Hagelgans A, Gussakovsky E, Kostka H, Paley EL, Siegert G. Differential expression of secretory phospholipases A2 in normal and malignant prostate cell lines: regulation by cytokines, cell signaling pathways, and epigenetic mechanisms. Neoplasia. 2008;10(3):279-86.

4. Thirkettle S, Decock J, Arnold H, Pennington CJ, Jaworski DM, Edwards DR. Matrix metalloproteinase 8 (collagenase 2) induces the expression of interleukins 6 and 8 in breast cancer cells. The Journal of biological chemistry. 2013;288(23):16282-94.

5. Pellinen T, Rantala JK, Arjonen A, Mpindi JP, Kallioniemi O, Ivaska J. A functional genetic screen reveals new regulators of beta1-integrin activity. J Cell Sci. 2012;125(Pt 3):649-61.

6. Laubli H, Borsig L. Selectins as mediators of lung metastasis. Cancer Microenviron. 2010;3(1):97-105.

7. Gomes IM, Maia CJ, Santos CR. STEAP proteins: from structure to applications in cancer therapy. Molecular cancer research : MCR. 2012;10(5):573-87.

8. Raccosta L, Fontana R, Maggioni D, Lanterna C, Villablanca EJ, Paniccia A, et al. The oxysterol-CXCR2 axis plays a key role in the recruitment of tumor-promoting neutrophils. The Journal of experimental medicine. 2013;210(9):1711-28.

9. Sharma B, Nawandar DM, Nannuru KC, Varney ML, Singh RK. Targeting CXCR2 enhances chemotherapeutic response, inhibits mammary tumor growth, angiogenesis, and lung metastasis. Mol Cancer Ther. 2013;12(5):799-808.

10. Rivas CI, Vera JC, Delgado-Lopez F, Heaney ML, Guaiquil VH, Zhang RH, et al. Expression of granulocyte-macrophage colony-stimulating factor receptors in human prostate cancer. Blood. 1998;91(3):1037-43.

11. Chen Z, Yang Y, Xiao Y, Zhao J. [Expression and significance of granulocyte-macrophage colony-stimulating factor receptors in human prostate cancer]. Zhonghua nan ke xue = National journal of andrology. 2004;10(9):655-7.

12. Liebscher CA, Prinzler J, Sinn BV, Budczies J, Denkert C, Noske A, et al. Aldehyde dehydrogenase 1/epidermal growth factor receptor coexpression is characteristic of a highly aggressive, poor-prognosis subgroup of high-grade serous ovarian carcinoma. Hum Pathol. 2013;44(8):1465-71.

13. Ohi Y, Umekita Y, Yoshioka T, Souda M, Rai Y, Sagara Y, et al. Aldehyde dehydrogenase 1 expression predicts poor prognosis in triple-negative breast cancer. Histopathology. 2011;59(4):776-80.

14. Kitamura K, Seike M, Okano T, Matsuda K, Miyanaga A, Mizutani H, et al. MiR-134/487b/655 Cluster Regulates TGF-beta-Induced Epithelial-Mesenchymal Transition and Drug Resistance to Gefitinib by Targeting MAGI2 in Lung Adenocarcinoma Cells. Mol Cancer Ther. 2014;13(2):444-53.

15. Luo Y, Kong F, Wang Z, Chen D, Liu Q, Wang T, et al. Loss of ASAP3 destabilizes cytoskeletal protein ACTG1 to suppress cancer cell migration. Mol Med Rep. 2014;9(2):387-94.

16. Dobrenis K, Gauthier LR, Barroca V, Magnon C. Granulocyte colony-stimulating factor off-target effect on nerve outgrowth promotes prostate cancer development. International journal of cancer Journal international du cancer. 2015;136(4):982-8.

17. Wang J, Ikeda R, Che XF, Ooyama A, Yamamoto M, Furukawa T, et al. VEGF expression is augmented by hypoxiainduced PGIS in human fibroblasts. International journal of oncology. 2013;43(3):746-54.

18. Kato T, Fujita Y, Nakane K, Mizutani K, Terazawa R, Ehara H, et al. CCR1/CCL5 interaction promotes invasion of taxane-resistant PC3 prostate cancer cells by increasing secretion of MMPs 2/9 and by activating ERK and Rac signaling. Cytokine. 2013;64(1):251-7.

19. Stubke K, Wicklein D, Herich L, Schumacher U, Nehmann N. Selectin-deficiency reduces the number of spontaneous metastases in a xenograft model of human breast cancer. Cancer letters. 2012;321(1):89-99.

20. Richter U, Schroder C, Wicklein D, Lange T, Geleff S, Dippel V, et al. Adhesion of small cell lung cancer cells to E- and P-selectin under physiological flow conditions: implications for metastasis formation. Histochem Cell Biol. 2011;135(5):499-512.

21. Kohler S, Ullrich S, Richter U, Schumacher U. E-/P-selectins and colon carcinoma metastasis: first in vivo evidence for their crucial role in a clinically relevant model of spontaneous metastasis formation in the lung. British journal of cancer. 2010;102(3):602-9.

22. Hu Y, Wu G, Rusch M, Lukes L, Buetow KH, Zhang J, et al. Integrated cross-species transcriptional network analysis of metastatic susceptibility. Proceedings of the National Academy of Sciences of the United States of America. 2012;109(8):3184-9.

23. Chigurupati S, Kulkarni T, Thomas S, Shah G. Calcitonin stimulates multiple stages of angiogenesis by directly acting on endothelial cells. Cancer research. 2005;65(18):8519-29.

24. Ferraro A, Schepis F, Leone V, Federico A, Borbone E, Pallante P, et al. Tumor suppressor role of the CL2/DRO1/CCDC80 gene in thyroid carcinogenesis. J Clin Endocrinol Metab. 2013;98(7):2834-43.

25. Lichao S, Liang P, Chunguang G, Fang L, Zhihua Y, Yuliang R. Overexpression of PTGIS could predict liver metastasis and is correlated with poor prognosis in colon cancer patients. Pathol Oncol Res. 2012;18(3):563-9.

26. Wang H, Fang R, Wang XF, Zhang F, Chen DY, Zhou B, et al. Stabilization of Snail through AKT/GSK-3beta signaling pathway is required for TNF-alpha-induced epithelial-mesenchymal transition in prostate cancer PC3 cells. European journal of pharmacology. 2013;714(1-3):48-55.

27. Geering K, Beguin P, Garty H, Karlish S, Fuzesi M, Horisberger JD, et al. FXYD proteins: new tissue- and isoform-specific regulators of Na,K-ATPase. Annals of the New York Academy of Sciences. 2003;986:388-94.

28. Nystedt J, Anderson H, Hirvonen T, Impola U, Jaatinen T, Heiskanen A, et al. Human CMP-N-acetylneuraminic acid hydroxylase is a novel stem cell marker linked to stem cell-specific mechanisms. Stem Cells. 2010;28(2):258-67.

29. Paccez JD, Vasques GJ, Correa RG, Vasconcellos JF, Duncan K, Gu X, et al. The receptor tyrosine kinase Axl is an essential regulator of prostate cancer proliferation and tumor growth and represents a new therapeutic target. Oncogene. 2013;32(6):689-98.

30. Urdinguio RG, Fernandez AF, Moncada-Pazos A, Huidobro C, Rodriguez RM, Ferrero C, et al. Immune-dependent and independent antitumor activity of GM-CSF aberrantly expressed by mouse and human colorectal tumors. Cancer research. 2013;73(1):395-405.

31. Zhang J, Patel L, Pienta KJ. CC chemokine ligand 2 (CCL2) promotes prostate cancer tumorigenesis and metastasis. Cytokine & growth factor reviews. 2010;21(1):41-8.

32. Okada T, Maeda A, Iwamatsu A, Gotoh K, Kurosaki T. BCAP: the tyrosine kinase substrate that connects B cell receptor to phosphoinositide 3-kinase activation. Immunity. 2000;13(6):817-27.

33. Koutros S, Schumacher FR, Hayes RB, Ma J, Huang WY, Albanes D, et al. Pooled analysis of phosphatidylinositol 3-kinase pathway variants and risk of prostate cancer. Cancer research. 2010;70(6):2389-96.

34. Devilard E, Bladou F, Ramuz O, Karsenty G, Dales JP, Gravis G, et al. FGFR1 and WT1 are markers of human prostate cancer progression. BMC cancer. 2006;6:272.

35. Brett A, Pandey S, Fraizer G. The Wilms' tumor gene (WT1) regulates E-cadherin expression and migration of prostate cancer cells. Molecular cancer. 2013;12:3.

36. El-Haibi CP, Singh R, Gupta P, Sharma PK, Greenleaf KN, Singh S, et al. Antibody Microarray Analysis of Signaling Networks Regulated by Cxcl13 and Cxcr5 in Prostate Cancer. J Proteomics Bioinform. 2012;5(8):177-84.

37. Li X, Sterling JA, Fan KH, Vessella RL, Shyr Y, Hayward SW, et al. Loss of TGF-beta responsiveness in prostate stromal cells alters chemokine levels and facilitates the development of mixed osteoblastic/osteolytic bone lesions. Molecular cancer research : MCR. 2012;10(4):494-503.

38. Ross RW, Galsky MD, Scher HI, Magidson J, Wassmann K, Lee GS, et al. A whole-blood RNA transcript-based prognostic model in men with castration-resistant prostate cancer: a prospective study. Lancet Oncol. 2012;13(11):1105-13.

39. Zhou X, Hao Q, Liao J, Zhang Q, Lu H. Ribosomal protein S14 unties the MDM2-p53 loop upon ribosomal stress. Oncogene. 2013;32(3):388-96.

40. Qin J, Xie Y, Wang B, Hoshino M, Wolff DW, Zhao J, et al. Upregulation of PIP3-dependent Rac exchanger 1 (P-Rex1) promotes prostate cancer metastasis. Oncogene. 2009;28(16):1853-63.

41. Hou Q, Tan HT, Lim KH, Lim TK, Khoo A, Tan IB, et al. Identification and functional validation of caldesmon as a potential gastric cancer metastasis-associated protein. Journal of proteome research. 2013;12(2):980-90.

42. Proost P, Loos T, Mortier A, Schutyser E, Gouwy M, Noppen S, et al. Citrullination of CXCL8 by peptidylarginine deiminase alters receptor usage, prevents proteolysis, and dampens tissue inflammation. The Journal of experimental medicine. 2008;205(9):2085-97.

43. Chu K, Cheng CJ, Ye X, Lee YC, Zurita AJ, Chen DT, et al. Cadherin-11 promotes the metastasis of prostate cancer cells to bone. Molecular cancer research : MCR. 2008;6(8):1259-67.

44. Cheong SM, Choi H, Hong BS, Gho YS, Han JK. Dab2 is pivotal for endothelial cell migration by mediating VEGF expression in cancer cells. Exp Cell Res. 2012;318(5):550-7.

45. Zavodszky E, Vicinanza M, Rubinsztein DC. Biology and trafficking of ATG9 and ATG16L1, two proteins that regulate autophagosome formation. FEBS Lett. 2013;587(13):1988-96.

46. Yang MY, Lin PM, Liu YC, Hsiao HH, Yang WC, Hsu JF, et al. Induction of cellular senescence by doxorubicin is associated with upregulated miR-375 and induction of autophagy in K562 cells. PloS one. 2012;7(5):e37205.

47. Yin H, Lowery M, Glass J. In prostate cancer C/EBPalpha promotes cell growth by the loss of interactions with CDK2, CDK4, and E2F and by activation of AKT. Prostate. 2009;69(9):1001-16.

48. Aung CS, Hill MM, Bastiani M, Parton RG, Parat MO. PTRF-cavin-1 expression decreases the migration of PC3 prostate cancer cells: role of matrix metalloprotease 9. Eur J Cell Biol. 2011;90(2-3):136-42.

49. Nassar ZD, Hill MM, Parton RG, Parat MO. Caveola-forming proteins caveolin-1 and PTRF in prostate cancer. Nat Rev Urol. 2013;10(9):529-36.

50. Xia G, Kageyama Y, Hayashi T, Hyochi N, Kawakami S, Kihara K. Positive expression of HIF-2alpha/EPAS1 in invasive bladder cancer. Urology. 2002;59(5):774-8.

51. Bangoura G, Yang LY, Huang GW, Wang W. Expression of HIF-2alpha/EPAS1 in hepatocellular carcinoma. World J Gastroenterol. 2004;10(4):525-30.
