# Supplementary material for: Digitoxin Inhibits Epithelial-to-Mesenchymal-Transition in Hereditary Castration Resistant Prostate Cancer
Source: Front Oncol. 2019 Aug 2;9:630. doi: 10.3389/fonc.2019.00630 (PMC6687970; doi:10.3389/fonc.2019.00630)
Supplement: Supplementary file 3 [file Table_3.DOCX]

**Supplementary Table 3.**

**Relationships between microRNAs detected in rat as either rno or mmu sequences, and human microRNAs, given as hsa sequences.**

| ***mmu* or *rno*** | ***Sequence*** | ***hsa*** | ***Sequence*** |
| --- | --- | --- | --- |
| **miR-98**  **let-7a-5p** | mmu-miR-98-5p MIMAT0000545  U**GAGGUAG**UAAGUUGUAUUGUU  rno-miR-98-5p MIMAT0000819  U**GAGGUAG**UAAGUUGUAUUGUU  mmu-let-7a-5p MIMAT0000521  U**GAGGUAG**UAGGUUGUAUAGUU  rno-let-7a-5p MIMAT0000774  U**GAGGUAG**UAGGUUGUAUAGUU | **miR-98**  **let-7a-5p** | hsa-miR-98-5p MIMAT0000096  U**GAGGUAG**UAAGUUGUAUUGUU  hsa-let-7a-5p MIMAT0000062  U**GAGGUAG**UAGGUUGUAUAGUU |
| **miR-346** | mmu-miR-346-5p MIMAT0000597  U**GUCUGCC**CGAGUGCCUGCCUCU  rno-miR-346 MIMAT0000596  U**GUCUGCC**UGAGUGCCUGCCUCU | **miR-346** | hsa-miR-346 MIMAT0000773  U**GUCUGCC**CGCAUGCCUGCCUCU |
| **miR-448-3p** | mmu-miR-448-3p MIMAT0001533  U**UGCAUAU**GUAGGAUGUCCCAU  rno-miR-448-3p MIMAT0001534  U**UGCAUAU**GUAGGAUGUCCCA | **miR-448** | hsa-miR-448 MIMAT0001532  U**UGCAUAU**GUAGGAUGUCCCAU |
| **miR-211-5p**  **miR-204-5p** | mmu-miR-211-5p MIMAT0000668  U**UCCCUUU**GUCAUCCUUUGCCU  rno-miR-211-5p MIMAT0000882  U**UCCCUUU**GUCAUCCUUUGCCU  mmu-miR-204-5p MIMAT0000237  U**UCCCUUU**GUCAUCCUAUGCCU  rno-miR-204-5p MIMAT0000877  U**UCCCUUU**GUCAUCCUAUGCCU | **miR-211-5p**  **miR-204-5p** | hsa-miR-211-5p MIMAT0000268  U**UCCCUUU**GUCAUCCUUCGCCU  hsa-miR-204-5p MIMAT0000265  U**UCCCUUU**GUCAUCCUAUGCCU |
